# The role of the components of PM_2.5_ in the incidence of Alzheimer’s disease and related disorders

**DOI:** 10.1016/j.envint.2025.109539

**Published:** 2025-05-18

**Authors:** Haisu Zhang, Yifan Wang, Haomin Li, Qiao Zhu, Tszshan Ma, Yang Liu, Kyle Steenland

**Affiliations:** Rollins School of Public Health, Emory University, United States

**Keywords:** Alzheimer’s, Dementia, PM_2.5_, Components, Mixture analysis

## Abstract

**Background:**

The associations of PM_2.5_ mass and various adverse health outcomes have been widely investigated. However, fewer studies focused on the potential health impacts of PM_2.5_ components, especially for dementia and Alzheimer’s diseases (AD).

**Methods:**

We constructed a nationwide population-based open cohort study among Medicare beneficiaries aged 65 or older during 2000–2018. This dataset was linked with the predicted levels of 15 PM_2.5_ components, including 5 major mass contributors (EC, OC, ${\text{NH}}_{4}^{+}$, ${\text{NO}}_{3}^{-}$, ${\text{SO}}_{4}^{2-}$) and 10 trace elements (Br, Ca, Cu, Fe, K, Ni, Pb, Si, V, Zn) across contiguous U.S. territory. Data were aggregated by ZIP code, calendar year and individual level demographics. Two mixture analysis methods, weighted quantile sum regression (WQS) and quantile g-computation (qgcomp), were used with quasi-Poisson models to analyze the health effects of the total mixture of PM_2.5_ components on dementia and AD, as well as the relative contribution of individual components.

**Results:**

Exposure to PM_2.5_ components over the previous 5 years was significantly associated with increased risks of both dementia and AD, with stronger associations observed for AD. ${\text{SO}}_{4}^{2-}$, OC, Cu were identified as major contributors to the combined positive association of the mixture from both WQS and qgcomp models.

**Conclusion:**

We found positive associations between the 15 PM_2.5_ components and the incidence of dementia and AD. Our findings suggest that reducing PM_2.5_ emissions from traffic and fossil fuel combustion could help mitigate the growing burden of dementia and Alzheimer’s disease.

## 1. Introduction

In 2019, the estimated number of dementia patients worldwide exceeded 55 million, with societal costs surpassing 1,300 billion US dollars (Wimo et al., 2023). In the United States alone, dementia affected more than 6.7 million Americans in 2023, and projections indicate 14 million cases by 2060 (Matthews et al., 2019). Alzheimer’s disease (AD) is the most common disease that leads to dementia and was the 6th leading cause of death in the United States (Heron, 2019). Given the absence of a cure for AD and related dementias (ADRD), a promising strategy to alleviate the health burden associated with these conditions is the identification and control of potential modifiable risk factors.

Growing evidence supports a relationship between air pollution and dementia, particularly regarding particulate matter with diameters less than 2.5 μm (PM_2.5_). Epidemiological studies conducted globally have consistently reported a positive association between long-term exposure to PM_2.5_ mass and the risk of dementia (Mortamais et al., 2021; Kriit et al., 2021; Lee et al., 2019; Shi et al., 2021; Zhang et al., 2023).

The complex composition of PM_2.5_ can benefit from a detailed examination, as the pathogenic effects of PM_2.5_ are not precisely elucidated when treated as a whole. Health studies at the component level are needed to quantify the relative hazards of specific components, providing actionable recommendations to policymakers for targeted pollution control. Numerous investigations have revealed positive long-term and short-term associations between PM_2.5_ components and various health outcomes, including non-accidental mortality (Yang et al., 2020; Chen et al., 2021; Danesh Yazdi et al., 2024; Kazemiparkouhi et al., 2022), cardiovascular diseases (Du et al., 2021; Liu et al., 2021), and respiratory diseases (Wu et al., 2021; Yang et al., 2021). However, few studies have investigated the health effects of PM_2.5_ components on dementia using a mixture analysis approach.

Another important limitation of past studies on the health effects of PM_2.5_ components is the lack of high-resolution exposure data. While some previous studies have relied on data from ground-based monitoring stations (Chung et al., 2015; Son et al., 2012), such data often fails to capture the potentially greater spatial heterogeneity of some components compared to PM_2.5_ mass. Moreover, the relatively limited number of monitoring stations for specific PM_2.5_ components has limited the scope of these studies. To cope with these challenges, many studies started to use data from exposure prediction models as an alternative (Yang et al., 2020; Du et al., 2021). Such models can achieve higher spatial resolution and good prediction accuracies, allowing for better estimation of their associations with adverse health outcomes.

A significant challenge in exploring potential relationships between PM_2.5_ components and adverse health outcomes is the observed high correlation among different particle components, as documented in previous studies (Chung et al., 2015; Chen et al., 2017; Bell Michelle et al., 2007). Our previous study evaluated 5 major PM_2.5_ components (EC, OC, ${\text{NH}}_{4}^{+}$, ${\text{NO}}_{3}^{-}$, ${\text{SO}}_{4}^{2-}$) and showed positive associations with AD and ADRD through single pollutant models (Shi et al., 2023). Here, we extend that research to incorporate the high-resolution data of 15 PM_2.5_ components and utilize two mixture analysis methods, which can control for component correlations: weighted quantile sum regression and quantile g-computation, to derive more robust and comprehensive estimates on the relative toxicity of these components. The aim of this research is to more accurately estimate the joint effects of PM_2.5_ mixture on dementia and Alzheimer’s disease, and identify the most important components within the mixture.

## 2. Methods

### 2.1. Study population

Two nationwide, privacy-protected and publicly available databases from the Centers for Medicare and Medicaid Services (CMS), including the Medicare denominator file and the Medicare Chronic Conditions Warehouse (CCW), were analyzed in this study. The denominator file contains enrollment records for each Medicare beneficiary, including demographics, Medicaid insurance status (a proxy for SES), the date of death, and ZIP code of residence, which were updated annually. The CCW claims data include predefined indicators for chronic conditions, based on ICD codes from health care providers, among the fee-for-service (FFS) Medicare beneficiaries and provides the date of the first occurrence with a diagnosis code for a specific condition.

We constructed separate cohorts for all-cause dementia and AD with the records from 2000 to 2018. For each cohort, we further required a “clean” period of 5 years after enrollment, during which the eligible beneficiaries could not have diagnosis codes for dementia or AD. For example, a beneficiary entering Medicare in 2003 would be required to be dementia-free by 2008; and the follow-up would only begin in 2008. The removal of potentially prevalent cases during the first 5 years of follow up was done to estimate incidence rather than prevalence effect measures. Overall, the study subjects entered the cohort on January 1st of the year following the “clean” period and were followed until the first diagnosis of the outcome of interest, death, or end of follow-up. All subjects were required to have continuous enrollment in Medicare Fee for Service for Parts A and B (doctors’ visits and hospitalizations) during follow-up to ensure consistent ascertainment of outcomes, as Medicare Advantage participants do not have data in the CCW file.

Our research is approved by Emory’s IRB (#STUDY00000316) and the Centers for Medicare & Medicaid Services (CMS) under the data use agreement (#RSCH-2020–55733). The Medicare dataset was stored and analyzed in Emory Rollins School secure cluster environment (HPC), with Health Insurance Portability and Accountability Act (HIPAA) compliance.

### 2.2. Outcome classification

There were two primary outcomes of interest in this study, all-cause dementia and AD. The diagnoses of dementia and AD were identified and recorded as two distinct indicators in the CCW dataset, which incorporated information across the available Medicare claims, including inpatient and outpatient claims, Carrier files (primarily doctor visits), skilled nursing facility, and home health-care claims. The accuracy of this algorithm has been proved in classifying diseases based on several validation studies (Taylor et al., 2002; Taylor et al., 2009). The ICD codes associated with the two indicators were presented in Table S1. Patients who had dementia diagnosis followed by a later diagnosis of AD were considered to have incident diagnosis of AD at the time of their first dementia diagnosis.

### 2.3. Exposure assessment

15 PM_2.5_ components were explored in this study, including 5 major mass contributors: elemental carbon (EC), organic carbon (OC), nitrate $\left({\text{NO}}_{3}^{-}\right)$, ammonium $\left({\text{NH}}_{4}^{+}\right)$, sulfate $\left({\text{SO}}_{4}^{2-}\right)$, which will be referred to as “major components” afterwards, and 10 trace elements: zinc (Zn) vanadium (V), silicon (Si), lead (Pb), nickel (Ni), potassium (K), iron (Fe), copper (Cu), calcium (Ca), bromine (Br). The annual mean predictions for the 15 PM_2.5_ components was estimated across the contiguous U.S. at a 50 m * 50 m spatial resolution in urban areas and a 1 km * 1 km spatial resolution in non-urban areas from 2000 to 2018 using super-learning and ensemble weighted averaging models. Details about the PM_2.5_ components dataset can be found elsewhere (Amini et al., 2022; Amini et al., 2023; Amini et al., 2023). Briefly, the training datasets consisted of data from 987 monitoring sites. 166 predictors were used for the super-learning and ensemble weighted-averaging models, including time and geography information, satellite observation data, meteorological data, emitting/surrogate of emission sources and other variables. These approaches achieved excellent model performance, with cross-validated R-square for major components ranging from 0.856 (OM) to 0.957 $\left({\text{SO}}_{4}^{2-}\right)$ and trace elements ranging from 0.79 (Cu) to 0.88 (Zn). We also obtained PM_2.5_ mass concentrations at 1 km * 1 km spatial resolution from 2000 to 2016 using the same super-learning and ensemble weighted-averaging models and reached a cross-validated R-square of 0.89 for annual predictions (Di et al., 2019). The PM_2.5_ components dataset and PM_2.5_ mass dataset have been widely used in previous epidemiological studies (Danesh Yazdi et al., 2024; Shi et al., 2023; Jin et al., 2022).

The gridded predictions for each PM_2.5_ component along with PM_2.5_ mass were then averaged at the ZIP code level with a population weighting approach. Within a ZIP code, the gridded population density value published on NASA SEDAC website were used as weights while calculating the spatial averages of PM_2.5_ components estimates (Center for International Earth Science Information Network, 2018). The moving average of up to 5 years prior to each calendar year were calculated by ZIP code, so that all people in the zip code have the same annual value for exposure. The ZIP code level 5 year moving average of exposures were then assigned to each Medicare beneficiary for each year, based on their residential ZIP code annually.

### 2.4. Covariates

Demographic characteristics at the individual level, including age, gender, and race, as well as Medicaid insurance status, were sourced from the Medicare denominator file. The age of beneficiaries was categorized into 4 groups: 65–75, 75–85, 85–95, 95+. The analytical models also integrated covariates at the neighborhood level, encompassing ZIP code-based socio-economic status variables such as population density, median household income, the percentage of the Black population, the percentage of the population residing in rental housing or apartments, and the percentage of the population with less than a high school education. Additionally, county-level factors, including behavioral risk factors such as smoking prevalence and mean body mass index, health care capacity variables like the number of hospitals and active medical doctors per 1,000 people, and a geographical region indicator of 5 U.S. regions (West, Southwest, Midwest, Northeast, Southeast) (National Geographic), were incorporated into the models. Those variables were included as presumed predictors of the outcome and as potential confounders if associated with air pollution levels. These variables were also used in our previous work by Shi et al. (2021), which provides comprehensive details and data sources for the covariates (Shi et al., 2021).

### 2.5. Statistical analysis

Our main analysis assessed the cumulative associations between 15 PM_2.5_ components and two primary outcomes, all-cause dementia and AD using two approaches, weighted quantile sum (WQS) regression models and quantile g-computation (qgcomp) models, with quasi-Poisson link function to account for potential over-dispersion.

The WQS models employ a two-step methodology to analyze the collective impact of exposure to a mixture of pollutants. Firstly, WQS calculates a composite index, representing the pollutant mixture, by deriving a weighted sum of quantiles for each pollutant, with optimal weights estimated by via regression of the studied outcome on the composite index in a training data set. Subsequently, the WQS model estimates the combined association between the pollutant mixture and the outcome through a multivariate regression model, utilizing the WQS term as the exposure metric. This approach provides a comprehensive understanding of the combined effects of multiple pollutants on a specific outcome, incorporating both the composition of the mixture and its impact (Carrico et al., 2015). One of the limitations for WQS model is that it requires directional homogeneity assumption, which means that all the exposures should have same direction associations with the outcome or have null associations.

The qgcomp models were developed as an extension of WQS regression models. Compared to WQS models, the qgcomp models do not require directional homogeneity and can incorporate both positive and negative associations between individual components of the mixture and the outcome. Qgcomp models use a marginal structural model to estimate the effect of the mixture. The qgcomp models can consistently estimate the effects of the exposures when WQS regression might be biased or inconsistent, but may also yield similar estimates with WQS regressions with large samples when its assumptions hold (Keil et al., 2020). We utilized both WQS and qgcomp models to compare the output from both models and serve as a cross-validation.

To fit the Poisson regression models used in the mixture analysis, individual-level data were aggregated into strata defined by ZIP code, calendar year, and individual-level covariates (age group, sex, race, and Medicaid status). However, due to computational limitations, the WQS models defined strata using only ZIP code and calendar year. In this case, individual-level covariates were summarized as the proportion of each category within each stratum (e.g., sex was represented as the percentage of males and females in each stratum).

For WQS, an index was estimated from ranking exposure concentrations in deciles. Then, the dataset was divided by 60:40 into the training dataset and validation dataset. 250 bootstrap samples were assigned for parameter estimation. We also tried different seeds to see if results were robust to random error. For qgcomp models. We also converted exposure concentrations into deciles. “qgcomp.noboot” procedure was used to estimate the associations. Both WQS and qgcomp models utilized quasi-Poisson regression, which accounts for any over-dispersion of Poisson assumptions. The observed number of cases in each stratum defined above was the outcome, and the predictors were deciles of the components and covariates. All the neighborhood level covariates were included as linear terms in models. We further calculated the component specific rate ratios using the weights of each component and the overall positive coefficient (and overall negative coefficient in qgcomp models). The weight of each component and the corresponding overall positive or negative coefficient were multiplied, and then exponentiated to derive rate ratios.

Several sensitivity analyses were also conducted. We fitted single and multi-pollutant (with all 15 components) stratified Cox proportional hazard models with a generalized estimating equation (GEE) to compare the traditional models with the mixture models. Single pollutant models and multi-pollutant models were fitted using individual level data, stratified by age group, race, sex, and Medicaid status, and controlled for same covariates in WQS and qgcomp models. We also tested the non-population-weighted predicted PM_2.5_ mass data from 2000 to 2018 to assess whether including the last two years of data and applying a population-weighting approach would influence the results of the Cox models. Besides the model types and specifications, we also explored the effect of movers in our study. Since beneficiaries might change their address during the follow-up period, utilizing the moving 5-year average exposures on ZIP code level could introduce exposure misclassification. Thus, we restricted the study population to beneficiaries that never change their address during the follow-up period for mixture models. Finally, we also conducted analyses using moving average PM_2.5_ component exposures up to 3 years prior to the current calendar year, comparing to the 5-year exposure window used in the main analyses.“.

All analyses were conducted in R software, version 4.2.2. WQS regression models were run with “gWQS” package and qgcomp models were run with “qgcomp” package (Renzetti et al., 2021; Keil, 2020). Statistical significance was determined by two-sided P < 0.05.

## 3. Results

### 3.1. Study population

**Fig. 1.**
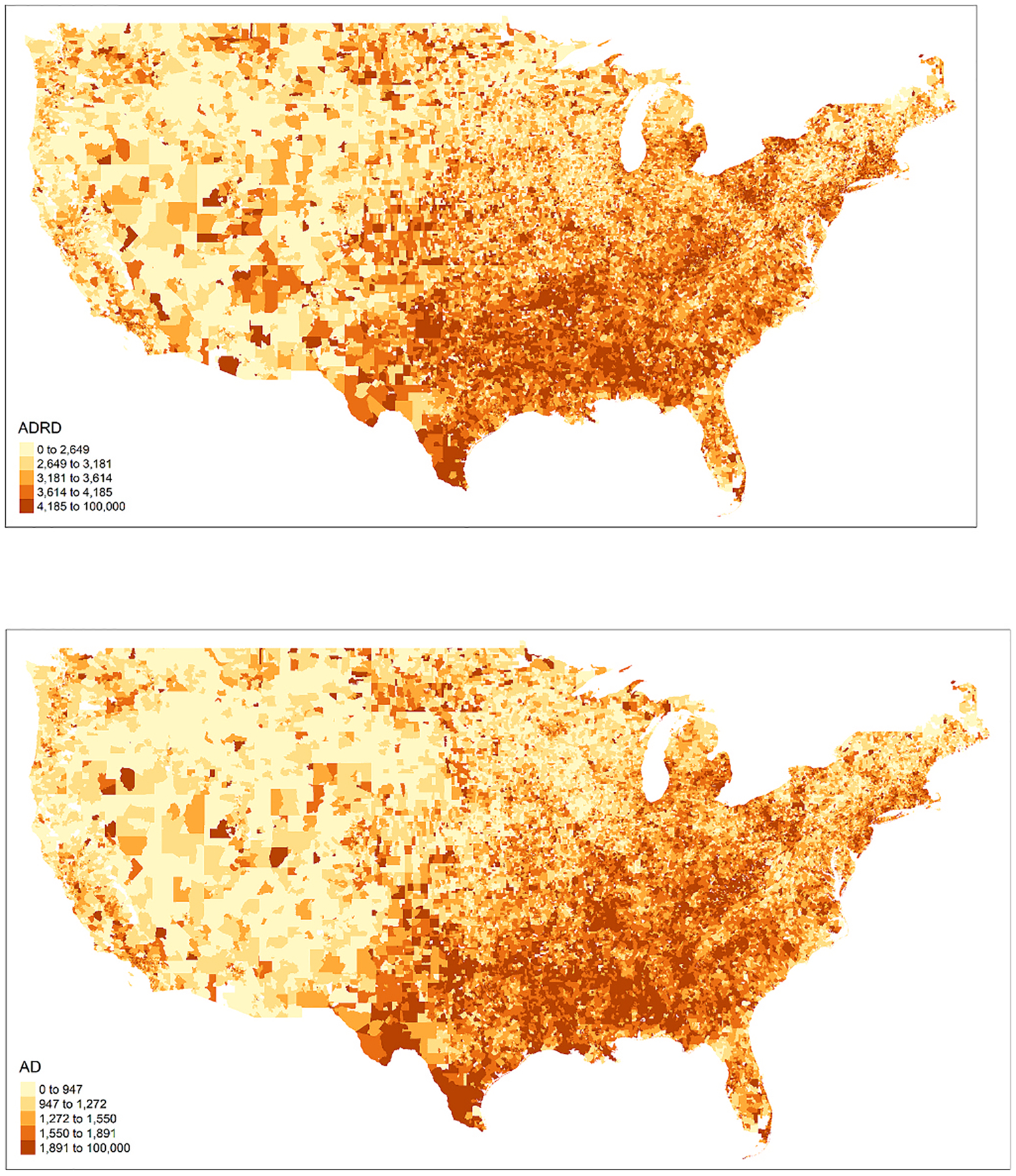
Distribution of Dementia / Alzheimer’s disease per 100,000 person-yr by ZIP code across the contiguous U.S.

Summary statistics of the dementia cohort and AD cohort can be found in Table 1. There were approximately 33.7 million beneficiaries in the dementia cohort and 34.6 million beneficiaries in the AD cohort. About 8.4 million (25.0 %) individuals developed dementia and 3.8 million (11.1 %) individuals developed AD. Most of the individuals in both cohorts were female (~57 %), White (~88 %), and not eligible for Medicaid insurance (~90 %). Fig. 1. shows the county-level incident diagnoses of dementia and AD per 100,000 Medicare beneficiaries in United States from 2000 to 2018. A relatively higher incident diagnoses of both dementia and AD were observed in the South and Southeast U.S.

### 3.2. Air pollution levels

The annual mean PM_2.5_ mass concentration (available during 2000–2016) was 9.87 μg/m^3^, which was lower than the EPA primary standard level during that period, 12.0 μg/m^3^, with an interquartile range (IQR) of 4.18 μg/m^3^. The mean concentrations of PM_2.5_ components are listed in Table 2. ${\text{SO}}_{4}^{2-}$ and OC contributed the most towards the PM_2.5_ mass with mean concentrations of 1.80 μg/m^3^ and 1.56 μg/m^3^ respectively. Among trace elements, Si, Fe, Ca contributed the most, with mean concentrations at 93.01, 49.84, 41.54 pg/m3, respectively. Fig. S2 shows the correlation matrix across all 15 PM_2.5_ components. The absolute values of correlation coefficients between PM_2.5_ components ranged from 0.01 to 0.88. Several PM_2.5_ components were highly correlated, such as Cu and EC (Pearson correlation coefficient r = 0.80), ${\text{NH}}_{4}^{+}$ and ${\text{SO}}_{4}^{2-}$ (r = 0.88), Pb and Zn (r = 0.76).

### 3.3. Qgcomp and WQS models on 15 PM_2.5_ components

The cumulative association between PM_2.5_ components and dementia/AD were summarized in Table 3. From the qgcomp models, the rate ratio for dementia was 1.027 (95 % CI: 1.025–1.028, per 1 decile increase in all PM_2.5_ components), while AD showed stronger associations with a rate ratio of 1.041 (95 % CI: 1.039–1.042). Similar but slightly larger estimates were obtained from WQS models, with the rate ratio for dementia at 1.029 (95 % CI: 1.028, 1.030) and AD at 1.048 (95 % CI: 1.047, 1.049) per 1 decile increase in all PM_2.5_ components.

**Fig. 2.**
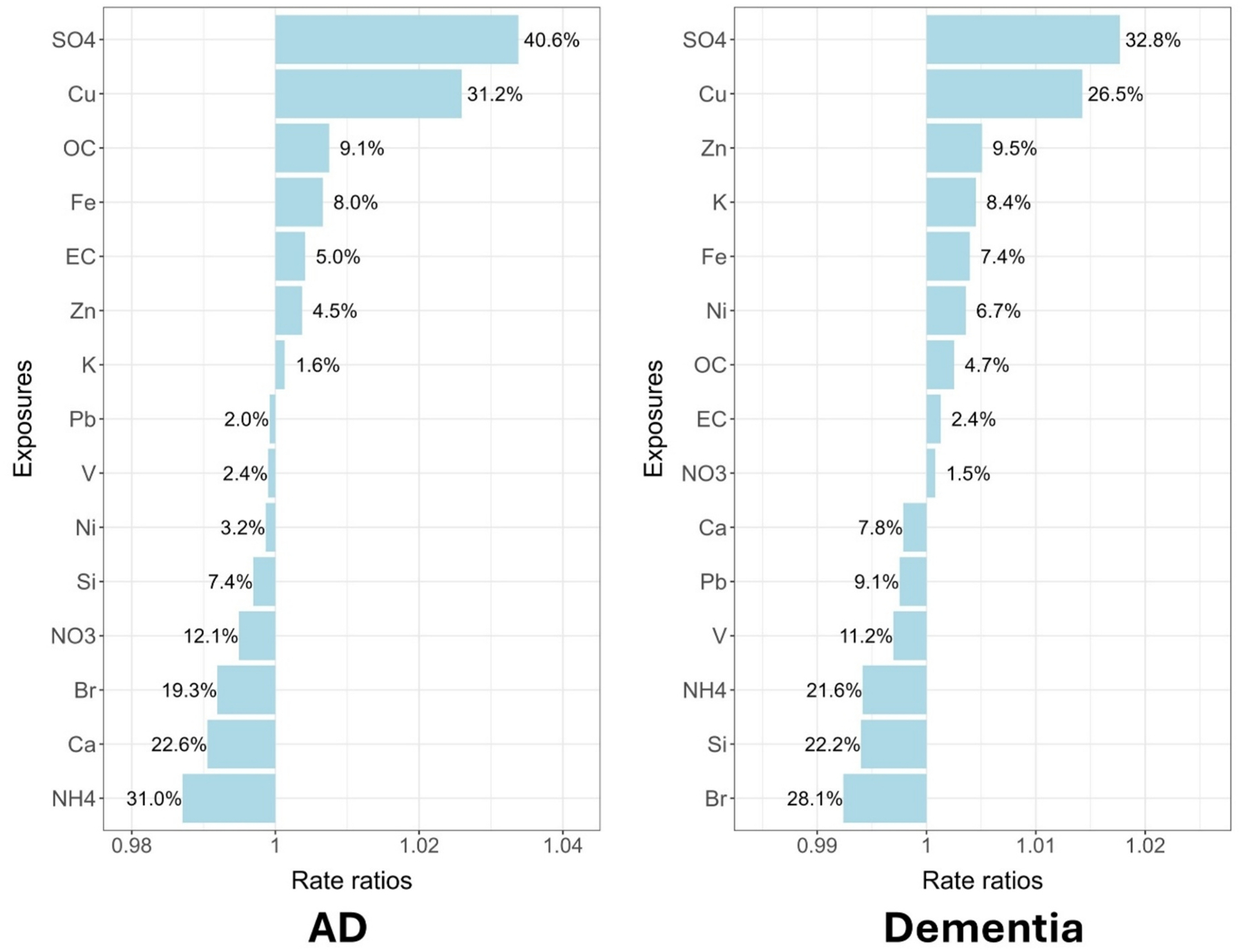
The weights (shown as percentages) and the calculated rate ratios (shown as bars) of each PM_2.5_ component for Alzheimer’s diseases / dementia from qgcomp models.

**Fig. 3.**
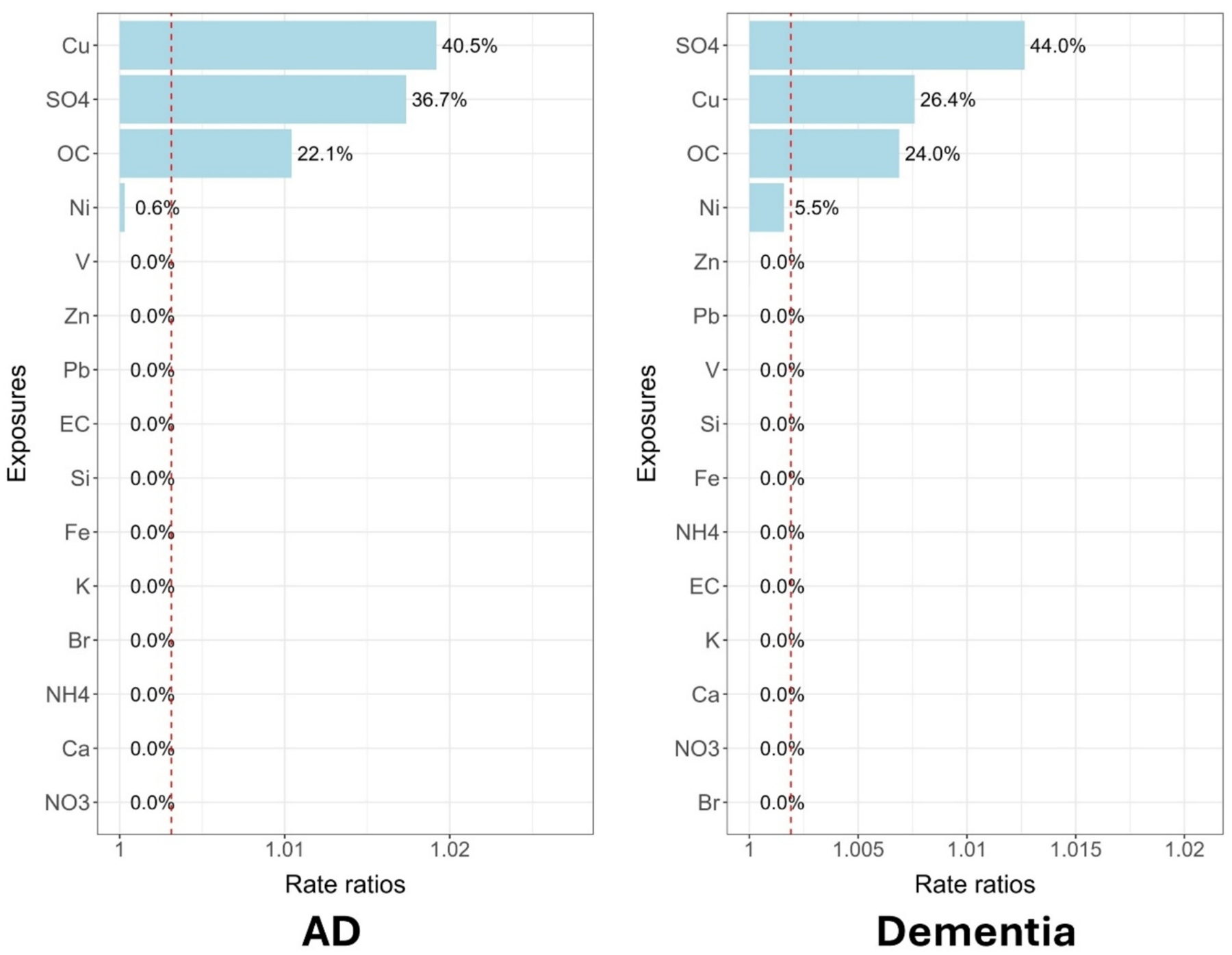
The weights (shown as percentages) and the calculated rate ratios (shown as bars) of each PM_2.5_ component for Alzheimer’s diseases / dementia from WQS models. Red dashed line marks the calculated rate ratio when weight = 1/15, which is the threshold for identifying potentially strong contributors among all PM_2.5_ components.

The weights and the calculated rate ratios for each PM_2.5_ component associated with dementia and AD in qgcomp and WQS models were shown in Figs. 2 and 3, respectively. Cu, ${\text{SO}}_{4}^{2-}$, and OC were among the highest weights for both dementia and AD in both qgcomp and WQS models. In addition, qgcomp model identified Fe for AD and Zn for dementia with relatively large positive weights; WQS model identified Ni for dementia for relatively large positive weights. The weights of other components were very small and negligible. The qgcomp models also identified some components with negative weights, but when converting to rate ratios, the magnitude were much smaller than components with positive weights.

### 3.4. Total PM_2.5_ mass and single and multiple component Cox models

The total PM_2.5_ mass and single PM_2.5_ component results are presented in Fig. S4 and Table S3, while results of multi-component models are show in Fig. S5 and Table S3. With per IQR increase in PM2.5 mass, the hazard ratio was 1.099 (95 % CI: 1.091, 1.106) for dementia and 1.074 (1.069, 1.079) for AD. Such associations remain almost unchanged when using the non-population-weighted PM_2.5_ mass data from 2000 to 2018. PM_2.5_ components with highest weights in mixture models also showed relatively strong association in single pollutant models. The hazard ratios of dementia with per IQR increase in PM_2.5_ components were 1.057 (95 % CI: 1.052, 1.062) for Cu, 1.086 (95 % CI: 1.081, 1.091) for ${\text{SO}}_{4}^{2-}$, and 1.042 (95 % CI: 1.037, 1.047) for OC. Similar but stronger associations were found for AD with hazard ratios (HRs) of 1.073 (95 % CI: 1.067, 1.080) for Cu, 1.099 (95 % CI: 1.091, 1.107) for ${\text{SO}}_{4}^{2-}$, 1.066 (95 % CI: 1.059, 1.074) for OC.

For Zn, we observed higher HR = 1.052 (95 % CI: 1.048, 1.055) for dementia but smaller HR for AD (1.043, 95 % CI: 1.038, 1.048), which was similar to the results in mixture models that Zn had relatively large weight for dementia but much smaller for AD. For Fe, we observed HR = 1.033 (95 % CI: 1.028, 1.038) for dementia, and 1.051 (95 % CI: 1.045, 1.057) for AD, which also matched with the higher weights for AD identified in qgcomp models.

Additionally, the single pollutant models identified EC (HR = 1.065, 95 % CI: 1.060, 1.071), ${\text{NH}}_{4}^{+}$ (HR = 1.060, 95 % CI: 1.055, 1.065), Br (HR = 1.042, 95 % CI: 1.037, 1.047) with relatively strong association with dementia. Similar associations were observed for AD and such associations were stronger for EC and Br, but slightly weaker for ${\text{NH}}_{4}^{+}$.

Similar results were found for multi-pollutant models, although the strength of associations were generally diminished. Copper, zinc, sulfate, EC, and OC organic carbon were all significant predictors of both AD and dementia, while iron was a predictor of AD. Strongest associations were seen for copper, sulfate, and iron (iron only for AD), followed with weaker associations for EC and OC.

### 3.5. Sensitivity analysis

The sensitivity analyses results were presented in Table s6. When restricting the study population to non-movers (about 85 % of the total cohort), the estimated rate ratio per deciles was slightly smaller for dementia (RR = 1.022, 95 % CI: 1.020, 1.023) and AD (RR = 1.036, 95 % CI: 1.034, 1.038) in qgcomp models, compared to the results for the full cohort. Both estimates were slightly smaller than those from the full study population. The weights under non-mover scenario were presented in Fig. s6. Compared to the main results, ${\text{SO}}_{4}^{2-}$ still has the largest weights, while the weight for Cu decreased in qgcomp models for dementia. Additionally, the weights of Fe increased in qgcomp models, and WQS models identified Fe as significant contributor for dementia.

When we use different seeds to run WQS models, the rate ratios and identified major contributors among PM_2.5_ mixture remained stable. When changing the exposure window from 5 years to 3 years, the estimated rate ratio almost did not change. The weights under 3-year exposure window were presented in figure s7. For weights in qgcomp models, ${\text{SO}}_{4}^{2-}$ and Cu remained the highest contributors, while the weights of other components varied slightly. The weights of OC decreased but still positively contributed to the total effect. The weights in WQS models almost did not change.

## 4. Discussion

In this study, we found positive cumulative associations between the PM_2.5_ components and both dementia and AD in mixture analyses. Such positive associations persist across different modeling approaches and specifications. The associations for AD were generally stronger than those for dementia. In both WQS and qgcomp models, ${\text{SO}}_{4}^{2-}$, Cu and OC were identified as the main contributors to both dementia and Alzheimer’s disease. Those three components also showed positive associations in both single and multi-pollutant Cox models and remained as important contributors under most of the sensitivity analysis scenarios.

The positive cumulative associations between PM_2.5_ components and both AD and dementia are consistent with findings from our previous study on the Medicare beneficiaries, which utilized PM_2.5_ mass as the primary exposure, and many other epidemiological studies (Shi et al., 2021; Carey et al., 2018; Smargiassi et al., 2020; Cristaldi et al., 2022). Our previous study on PM2.5 mass and dementia with Medicare population uncovered that per IQR increase in PM2.5 mass was associated with 6 % increase in hazard of dementia (Shi et al., 2021). Another study in London on 130,978 elderly people also reported 7 % increase in hazard of dementia associated with per IQR increase in PM2.5 mass (Carey et al., 2018). The relatively weaker associations for dementia compared to AD might result from the broader spectrum of different mental disorders of dementia. Some of the non-AD disorders under dementia might have weaker association with PM_2.5_ exposures (Rhew et al., 2021).

The cumulative associations were much stronger when converted to per IQR increase in all components (RR = 1.222 for qgcomp model and 1.264 for WQS model) compared to that for total PM_2.5_ mass (HR = 1.099). Such differences could be due to the temporal and spatial variations in the composition of PM_2.5_. When the PM_2.5_ mass level changes, different components might not change in the same magnitude, potentially resulting in less strong overall health effects. In contrast, the mixture analysis the HRs for the mixture reflect a simultaneous increase for *all* components in the same magnitude, which might result in higher HRs than PM_2.5_ mass when both rescaled to per IQR increase. Such differences in magnitude were generally smaller when comparing the calculated component specific RRs from mixture analysis models and HRs from single and multi-pollutant models. Overall, we suggest that the interpretation of mixture model results should be within the context of PM_2.5_ mixtures, and the comparison with traditional Cox models should be made cautiously.

Cu, ${\text{SO}}_{4}^{2-}$ and OC were characterized as the most important contributing factors to incident diagnoses of AD and dementia among all the 15 PM_2.5_ components. Such results were also supported by the single pollutant Cox models. It is well-established that PM_2.5_ is small enough to be absorbed into blood circulation through inhalation, and can penetrate the blood–brain barrier (BBB) (You et al., 2022), which indicates that the components in the PM_2.5_ mixture could potentially participate in the metabolism of the nervous system. ${\text{SO}}_{4}^{2-}$ and organic carbon are two major PM_2.5_ components that contribute to large fractions in PM_2.5_ mass. Particulate ${\text{SO}}_{4}^{2-}$ mainly originates from the oxidation of sulfur dioxide (SO_2_) in the atmosphere (Zhang et al., 2015), which primarily originated from combustion of fossil fuel combustion, especially coal, and biomass burning (Huang et al., 2023; Ding et al., 2021; Zhong et al., 2020; Shen et al., 2021). While studies directly examining the effects of ${\text{SO}}_{4}^{2-}$ exposure on the nervous system are limited, ${\text{SO}}_{4}^{2-}$ might serve as a proxy for fossil fuel combustion and industrial emission sources. Co-emitted pollutants from those sources, such as volatile organic compounds (VOCs), have been associated with increased risk of AD and dementia through oxidative stress and neuroinflammation (Khan et al., 2021). While these co-emitted pollutants may differ from ${\text{SO}}_{4}^{2-}$ in terms of environmental fate and transport, it is possible that some health effects of ${\text{SO}}_{4}^{2-}$ found in our model reflect the influence of those co-emitted pollutants. Meanwhile, elevated level of ${\text{SO}}_{4}^{2-}$ could be associated with increased aerosol acidity, which enhances the solubility of metals, such as Cu (Fang et al., 2017). Such increase in soluble metal fractions in PM_2.5_ mixture could lead to stronger bioavailability and toxicity (Verma et al., 2015; Luo et al., 2024), which potentially contributing to a higher risk of dementia and AD. Continued efforts to reduce fossil fuel combustion and improve emission control technologies might play a critical role in mitigating the burden of AD and dementia.

In terms of OC, the exact compound and the emission sources were more complex. OC could be categorized into primary OC, which is mainly produced by vehicle emissions, industrial processes and wild-fires, etc., and secondary OC, which is formed through chemical reactions involving VOCs (Saarikoski et al., 2008). The neurotoxicity of different compounds in OC could differ a lot. For example, Polycyclic aromatic hydrocarbons (PAHs) and polychlorinated biphenyls (PCBs) in OC are highly toxic to nervous system through multiple possible pathways, such as oxidative stress, disruption of thyroid hormone signaling and altered neurotransmitter signaling (Kodavanti, 2005; Tang et al., 2003; Pessah et al., 2019). More research on the health effects of OC on nervous systems are warranted.

Cu along with Zn and Fe that were identified by qgcomp models are trace elements in PM_2.5_ mixtures. Although trace elements only account for a small part in PM_2.5_ mixtures, they could still have important health impacts. Previous research has shown that Cu could play an important role in the development and progression of neurodegenerative diseases (Mathys and White, 2017). Excessive exposure to Cu could result in the dysregulation of Cu homeostasis in brain tissues (Bulcke et al., 2017). Cu overload can cause oxidative stress which could further cause damage to brain cells (Scheiber et al., 2014). Fe is an essential cofactor for many proteins in brain, however, various studies have shown that the abnormally high concentrations of Fe in some brain tissues were associated with neurodegenerative disorders (Zecca et al., 2004; Connor et al., 1992). Elevated Fe levels in brain could contribute to β-amyloid (Aβ) dysfunction, formation of plaque, the hyperphosphorylation of tau protein, and neuronal cell death which are the classical features in the pathology of AD (Lane et al., 2018; Telling et al., 2017; Yamamoto et al., 2002). Zn is also an important element in neurons. Disruptions to Zn homeostasis has been associated with multiple nervous system disorders such as AD and Parkinson’s disease (Wang et al., 2023). Elevated Zn levels in brain tissue could also lead to the deposition of Aβ and overly phosphorylated tau protein, which contributed to AD (Brewer et al., 2010; Mocchegiani et al., 2005). Considering the plausible adverse health effects of Cu, Zn and Fe on AD and dementia, policies targeting the related emission sources are vital. For example, non-tailpipe emissions from brake and tire wear are significant contributors to the metal components in particulate matter, including Cu, Zn and Fe (Grigoratos and Martini, 2015; Lopez et al., 2023). With the implementation of mobile source regulations and the growing adoption of electric vehicles (EVs), significant reductions in tailpipe emissions have been achieved, and the relative contribution of non-tailpipe emissions has been increasing (Habre et al., 2021). Targeting the non-tailpipe emissions, EPA has introduced the Copper-free Brake Initiative since 2015 (Brake, 2015), and we call for more effort in the reduction of non-tailpipe emissions.

The qgcomp models also identified some components with negative weights. The components with negative weights were not necessarily protective for the outcomes on their own. It is possible that these components showed an antagonistic effect and weakened the hazardous impacts of other components in the mixture. Previous research reported the interaction effects between major air pollutants on health outcomes, such as the interaction between PM_2.5_ and NO_2_ (Mainka and Żak, 2022). However, research on the interaction in a mixture setting, such as PM_2.5_ components, remains limited. We encourage future studies to elucidate the complex relationships between PM_2.5_ components and health outcomes. Given that the overall mixture effects were positive and robust across all sensitivity analyses for both AD and dementia, we believe the focus should remain on the hazardous impacts of PM_2.5_ components.

While the epidemiological association between PM_2.5_ mass and dementia has been extensively studied, there are not many studies looking into component-specific associations, especially with the mixture analysis techniques. Our previous study revealed the potential effects of ${\text{SO}}_{4}^{2-}$ and black carbon (BC) on dementia with traditional Cox models (Shi et al., 2023). More studies that focus on the PM_2.5_ components or PM_2.5_ sources are warranted to better elucidate the health effects of different components in PM_2.5_ mixtures and contribute to targeted policies on PM_2.5_ sources.

There are several strengths in our study. First, our study utilized nationwide, population-based open cohort constructed from the Medicare database to explore the potential health effects of PM_2.5_ mixtures on dementia and AD. The large sample size provided us with strong power to identify the potential health effects. Second, we used mixture analysis techniques, qgcomp and WQS, to avoid the biases derived from collinearity in multi-pollutant models and address the issue of confounding from other PM_2.5_ components in single pollutant models. By comparing the results from mixture analysis methods and traditional methods, our study could provide a more solid insight of the associations between PM_2.5_ components and dementia. Third, our study used comprehensive Medicare claims with physician visits included, which could better capture the earlier diagnosed cases. Compared to other studies that used hospitalization records, our study could better identify incident cases.

There are also several limitations in our study. First, we used predicted PM_2.5_ components concentrations from machine learning models. Though the model performance was excellent, potential prediction errors still exist. Meanwhile, the linkage of exposure to Medicare beneficiaries was conducted on ZIP code level since we only had the residential ZIP code of each beneficiary enrolled in Medicare. Although we have used population-weighted exposures within each ZIP code, some exposure misclassification is inevitable. Second, we used AD and dementia diagnoses to approximate the incidence of diseases. Although the 5-year clean period could help eliminate some non-incident diagnoses, we could not directly obtain the incidence of AD or dementia in the present study. On the other hand, longitudinal data following large populations across time to include both disease onset and eventual diagnosis are rare, and many studies had to rely on a diagnosis date. Third, the qgcomp and WQS models were quantile-based models, and the overall estimates are based on each PM_2.5_ component increase by the same quantile. However, in real-life situations, different PM_2.5_ components rarely change in similar ways, which makes the overall estimates harder to interpret. Fourth, we did not try to account for the potential non-linear effects of PM_2.5_ components on AD or dementia. Our previous paper revealed that most of the major PM_2.5_ components had near linear relationship with AD and dementia in single pollutant Cox models (Shi et al., 2023). Future studies could try to explore the potential non-linear health effects of PM_2.5_ components in mixture analysis. Finally, we did not account for the potential outcome misclassification in this study. Since the progression of dementia and AD is a long process, the identification of incident cases was still challenging although we have used comprehensive claims and a 5-year clean period. Misdiagnoses of AD and other dementia has been reported among Medicare beneficiaries before (Taylor et al., 2009). Potential misclassification of AD outcome in our Medicare dataset was investigated previously by our group via two different methods in our previous study on the health effects of PM_2.5_ mass on AD and dementia, and revealed that such outcome misclassification would like to lead to some modest under-estimation of the true hazard ratios (Shi et al., 2021).

## 5. Conclusion

Our study revealed a strong positive association between the PM_2.5_ components mixture with both dementia and Alzheimer’s diseases. Cu, ${\text{SO}}_{4}^{2-}$ and OC were identified as potentially more important contributors among the 15 PM_2.5_ components. Our findings suggested that policies that target the reduction of ambient PM_2.5_ concentrations should continue to be implemented to reduce the burden of dementia and Alzheimer’s diseases in U.S. Reduction of PM_2.5_ emissions from traffic and fossil fuel combustion could help mitigate the growing burden of dementia and Alzheimer’s disease.

## Supplementary Material

1

Appendix A. Supplementary material

Supplementary data to this article can be found online at https://doi.org/10.1016/j.envint.2025.109539.

## Figures and Tables

**Table 1:** Descriptive statistics of the study population and area-level covariates among the dementia cohort and Alzheimer’s disease (AD) cohort.

	Alzheimer’s Diseases N (%)		Dementia N (%)	
Number of cases	2,997,902	(10.8)	6,801,584	(25.4)
Number of patients	27,763,593		26,745,676	
Total person-years	178,701,891		166,689,100	
Age at entry	75.4	(5.6)	75.2	(5.4)
Sex				
Male	11,676,349	(42.1)	11,302,092	(42.3)
Female	16,087,244	(57.9)	15,443,584	(57.7)
Race				
White	24,768,655	(89.2)	23,891,902	(89.3)
Black	1,861,196	(6.7)	1,765,910	(6.6)
Others	1,133,742	(4.1)	1,087,864	(4.1)
Dual eligibility (PY)*	19,347,604	(10.8)	16,136,507	(9.7)

* Percentages of dual eligibility are based on total person-years. Other percentages are based on number of patients.

**Table 2:** Summary statistics of PM_2.5_ component concentrations across study period.

Pollutants	IQR	Mean	SD	25th	Median	75th	95th	Max
EC (μg/m^3^)	0.31	0.44	0.26	0.26	0.36	0.57	0.94	2.33
${\text{NH}}_{4}^{+}$ (μg/m^3^)	0.65	0.73	0.45	0.37	0.63	1.02	1.60	2.84
${\text{NO}}_{3}^{-}$ (μg/m^3^)	0.71	0.92	0.56	0.50	0.77	1.22	2.00	4.83
OC (μg/m^3^)	0.75	1.56	0.64	1.12	1.44	1.87	2.75	7.14
${\text{SO}}_{4}^{2-}$ (μg/m^3^)	1.48	1.80	1.07	0.98	1.53	2.45	3.99	6.47
Br (pg/m^3^)	1.18	2.22	0.83	1.63	2.21	2.81	3.59	8.09
Ca (pg/m^3^)	27.67	41.54	25.66	24.59	34.61	52.26	86.72	326.35
Cu (pg/m^3^)	1.96	1.88	1.66	0.69	1.23	2.65	5.39	17.73
Fe (pg/m^3^)	31.98	49.84	28.43	30.59	42.39	62.57	106.99	241.28
K (pg/m^3^)	18.20	51.62	15.42	41.94	50.65	60.15	76.77	232.27
Ni (pg/m^3^)	0.53	0.45	0.49	0.11	0.33	0.63	1.26	6.23
Pb (pg/m^3^)	1.45	2.05	1.29	1.16	1.62	2.61	4.82	9.72
Si (pg/m^3^)	63.28	93.01	53.02	55.55	80.30	118.83	187.00	581.00
V (pg/m^3^)	0.59	0.61	0.73	0.17	0.35	0.76	2.15	9.33
Zn (pg/m^3^)	4.30	6.78	3.89	4.22	5.92	8.51	13.87	46.59
Total PM_2.5_ mass (μg/m^3^)	4.18	9.87	3.17	7.76	9.76	11.94	15.10	30.92

Note: PM_2.5_ mass concentrations are available from 2000 to 2016. Abbreviations: IQR: interquartile range. SD: Standard deviation. 25th: 25th percentile. 75th: 75th percentile. 95th: 95th percentile. ${\text{SO}}_{4}^{2-}$: sulfate; ${\text{NO}}_{3}^{-}$: nitrate; ${\text{NH}}_{4}^{+}$: ammonium; OC: organic carbon; EC: elemental carbon; Zn: zinc; V or vanadium; K: potassium; Si: silicon; Pb: lead; Ni: nickel; Fe: iron; Cu: copper; Ca: calcium; Br: bromine.

**Table 3:** Cumulative associations between PM_2.5_ components and Alzheimer’s diseases / dementia in rate ratios (RRs) estimated from both WQS and qgcomp models.

	WQS	qgcomp
Dementia	1.029 (1.028, 1.030)	1.027 (1.025, 1.028)
Alzheimer’s diseases	1.048 (1.047, 1.049)	1.041 (1.039, 1.042)

Note: The rate ratios are estimated with 1 decile increase in all PM_2.5_ components.

## Data Availability

I have included the data availability info in supplementary documents.
