# Supplementary material for: The role of the components of PM2.5 in the incidence of Alzheimer’s disease and related disorders
Source: Environ Int. Author manuscript; Available in PMC 2025 Jun 23. (PMC12185172; doi:10.1016/j.envint.2025.109539)
Supplement: 1 [file NIHMS2087677-supplement-1.docx]

The role of the components of PM_2.5_ in the incidence of Alzheimer’s disease and related disorders

Supplementary materials

Haisu Zhang^1^, Yifan Wang^1^, Haomin Li^1^, Qiao Zhu^1^, Tszshan Ma^1^, Yang Liu^1^, Kyle Steenland^1^

^1^ Rollins School of Public Health, Emory University

Figure S1: ZIP code level mean concentrations (μg/m^3^) of 15 PM_2.5_ components across 2000-2018


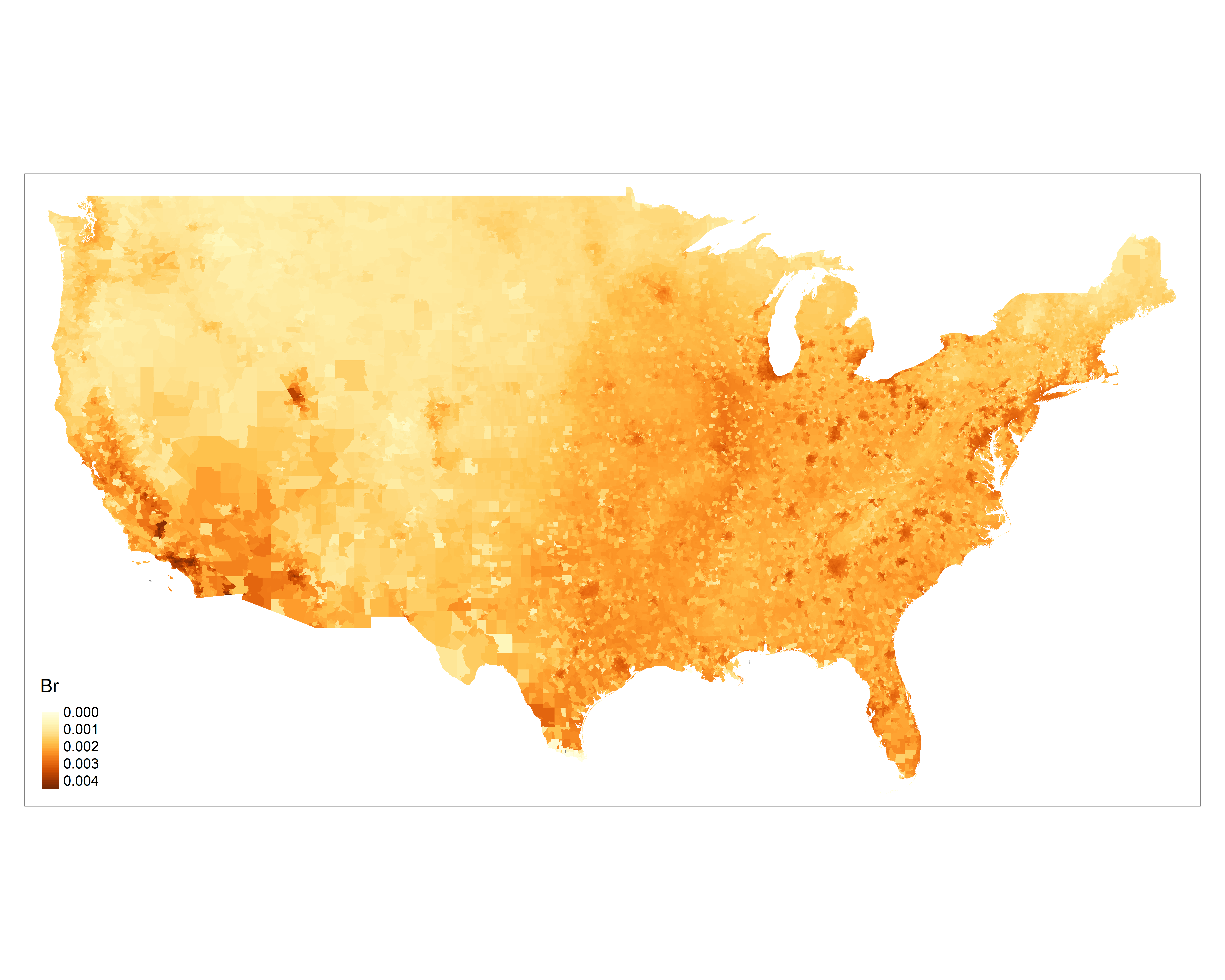

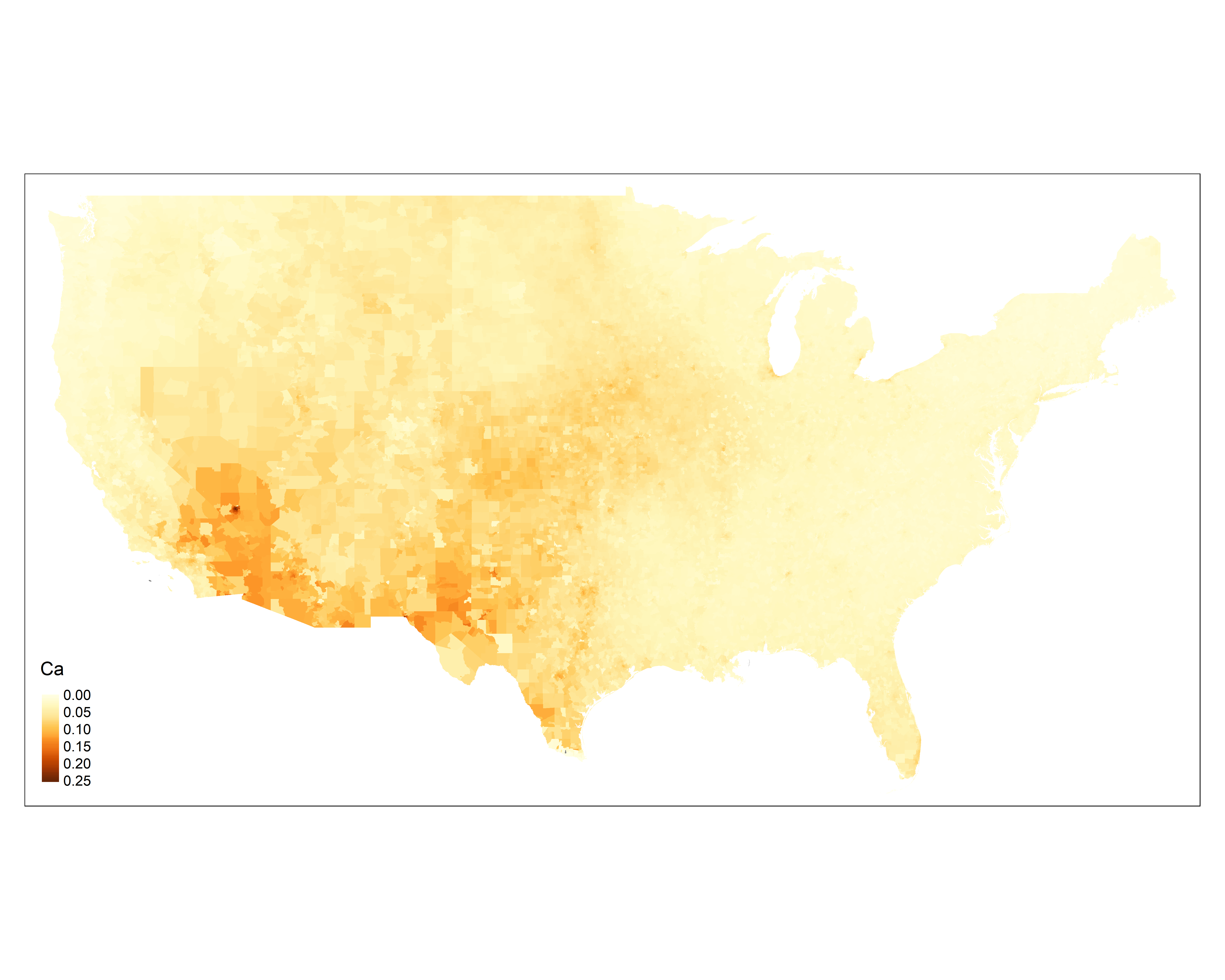

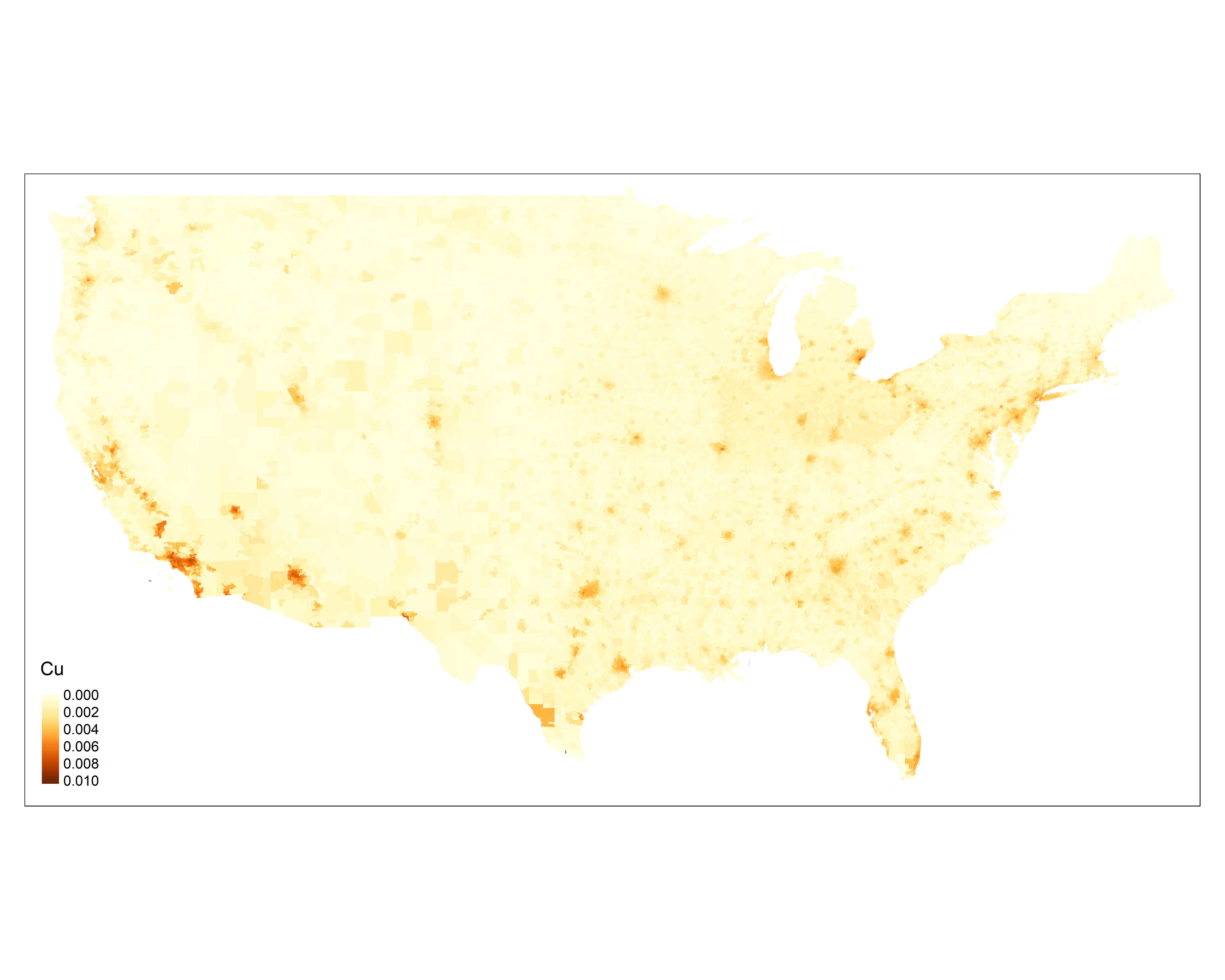

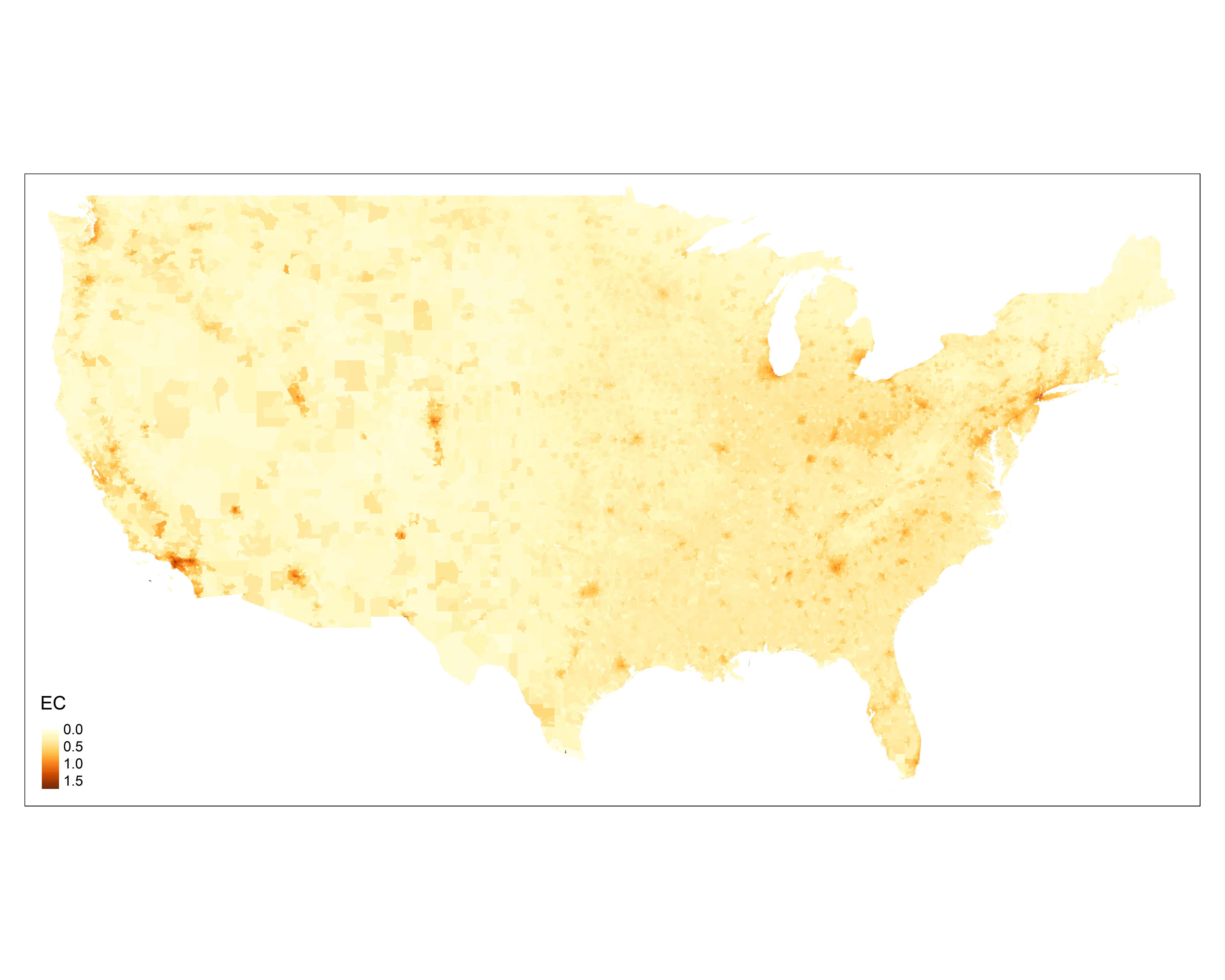

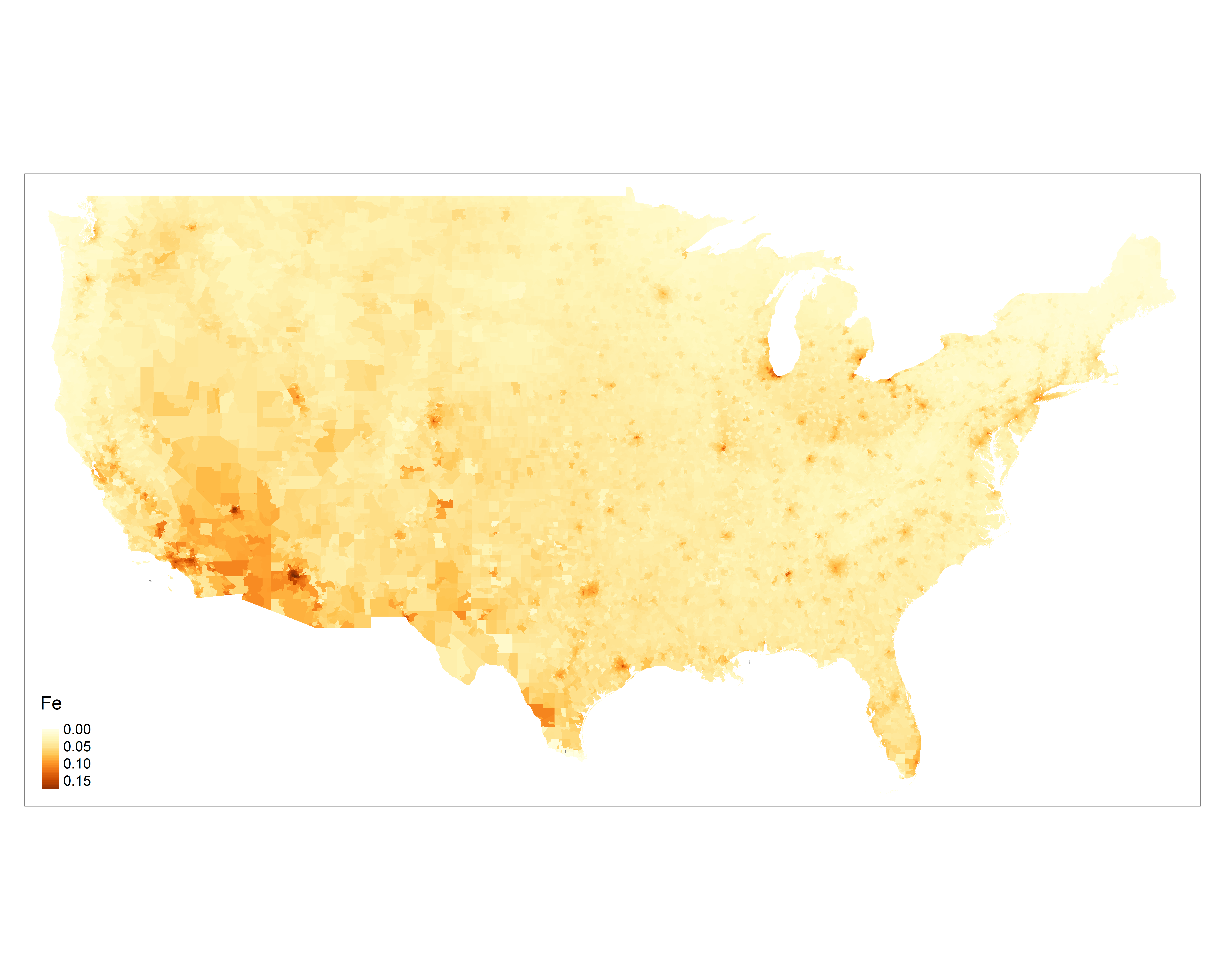

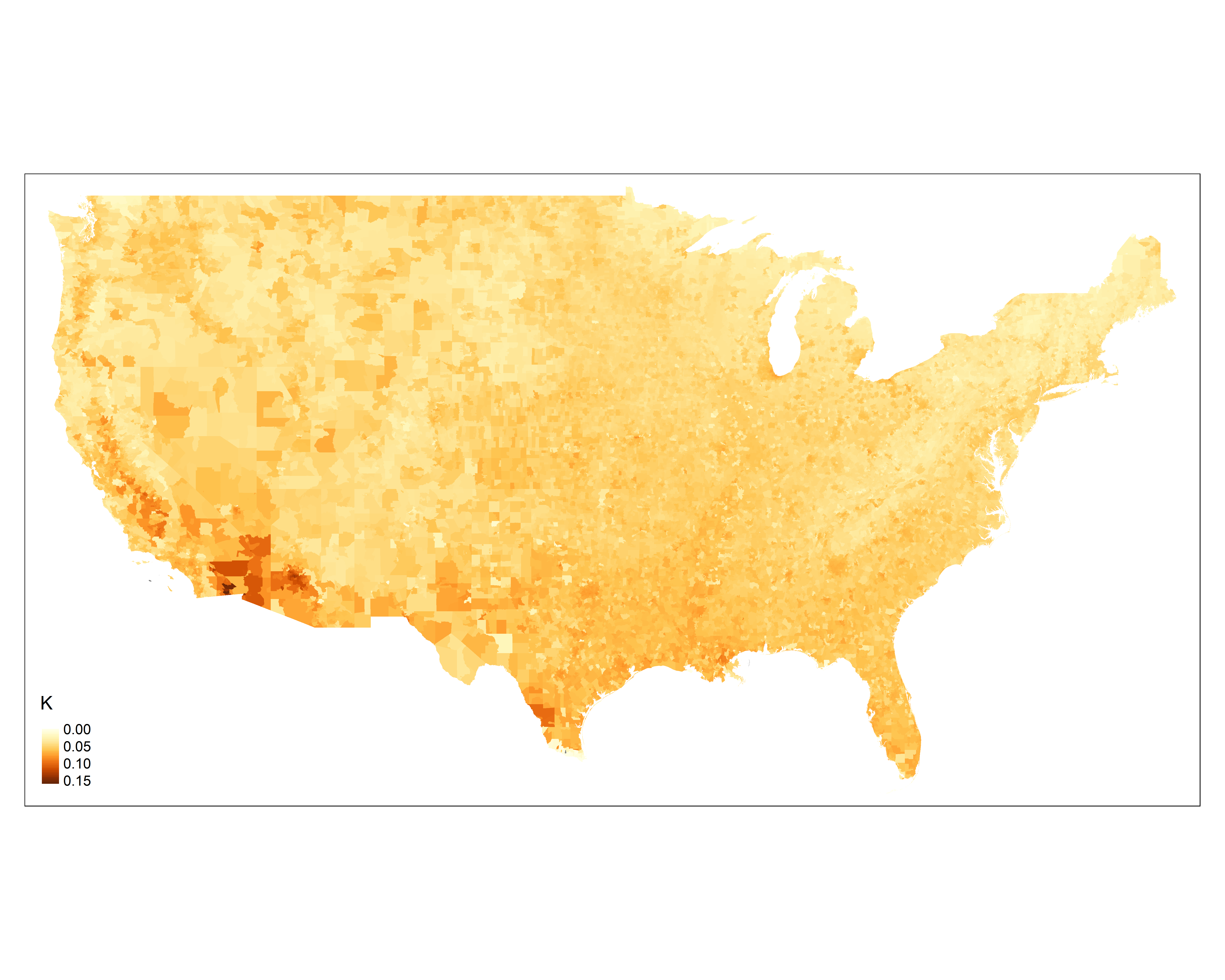

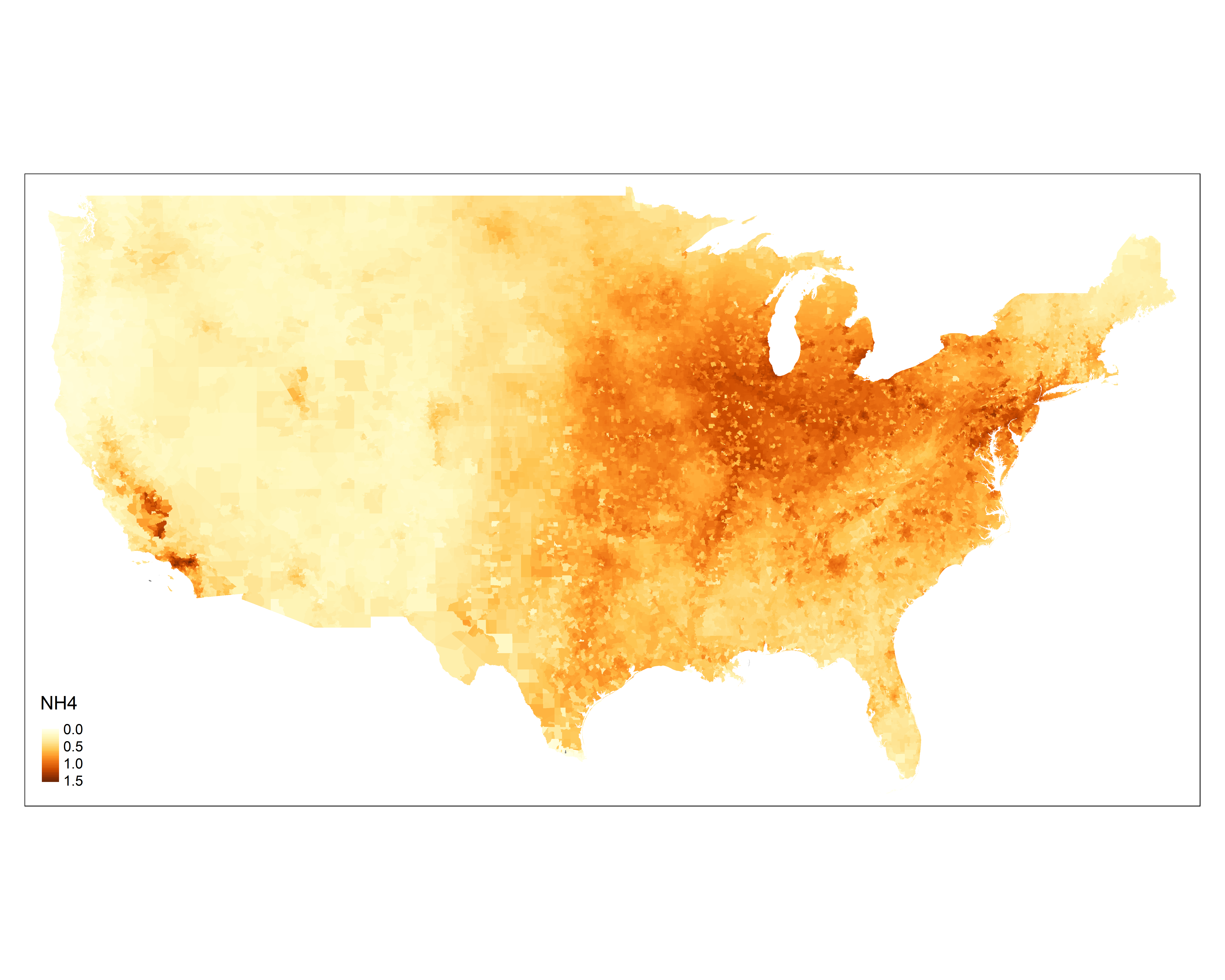

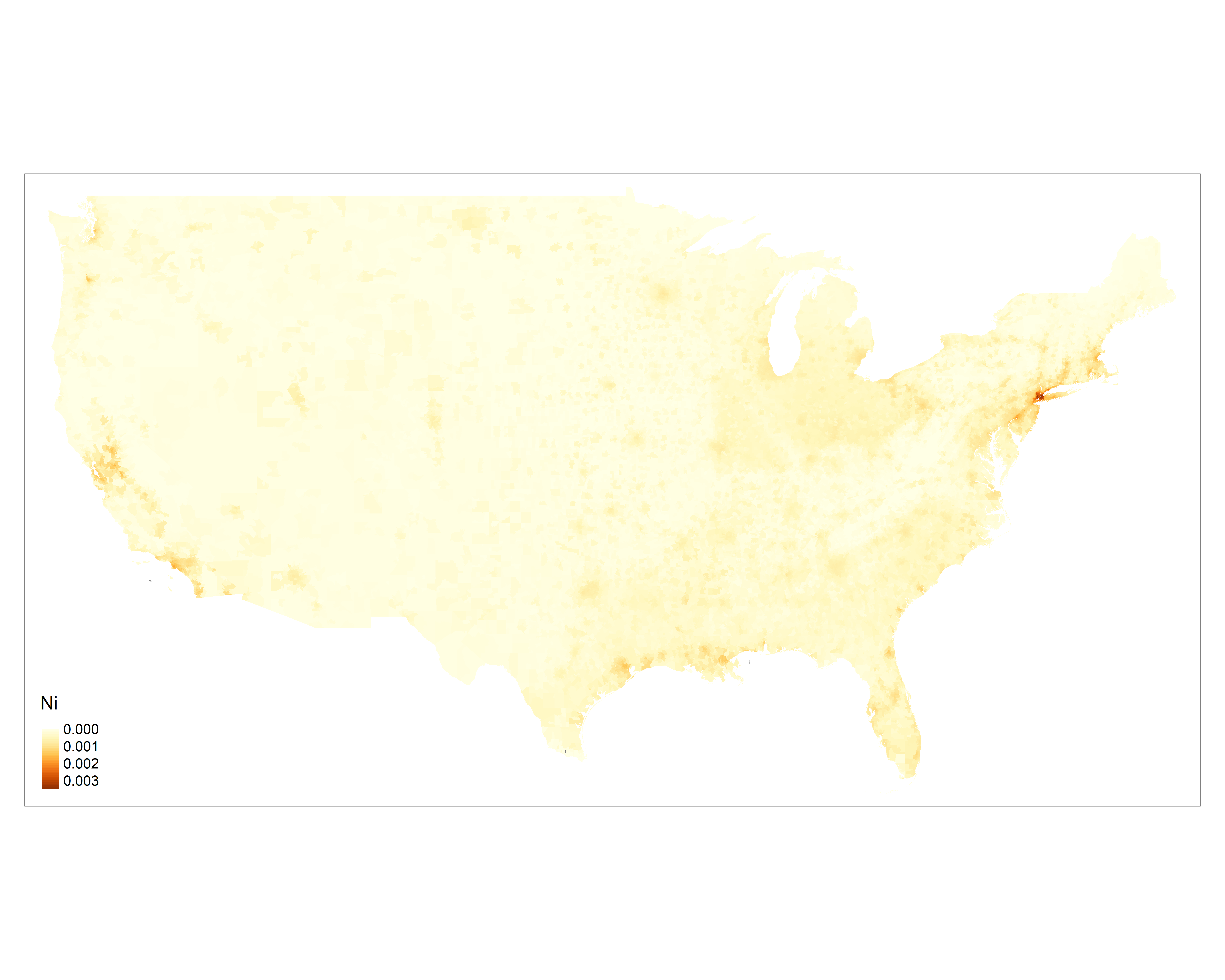

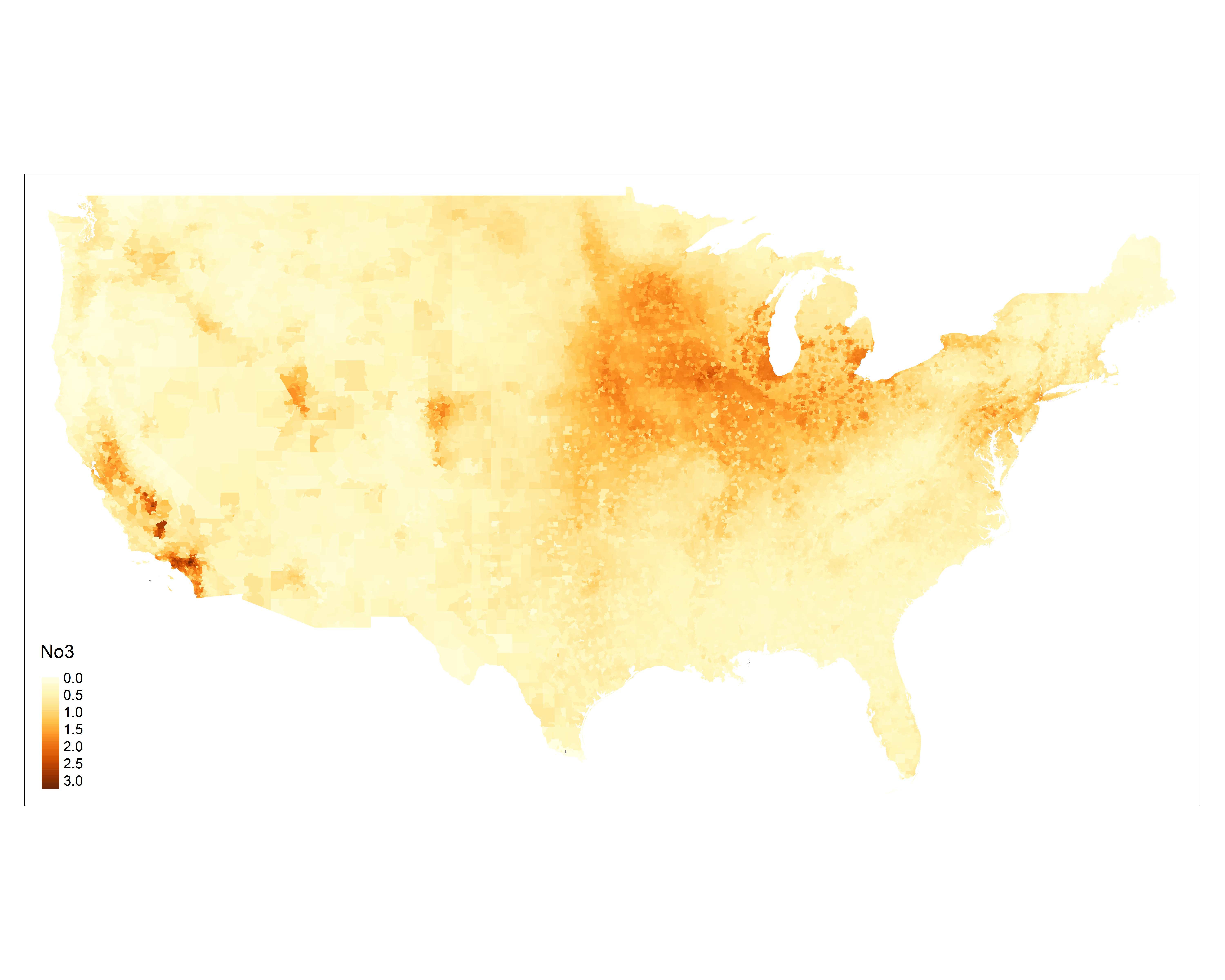

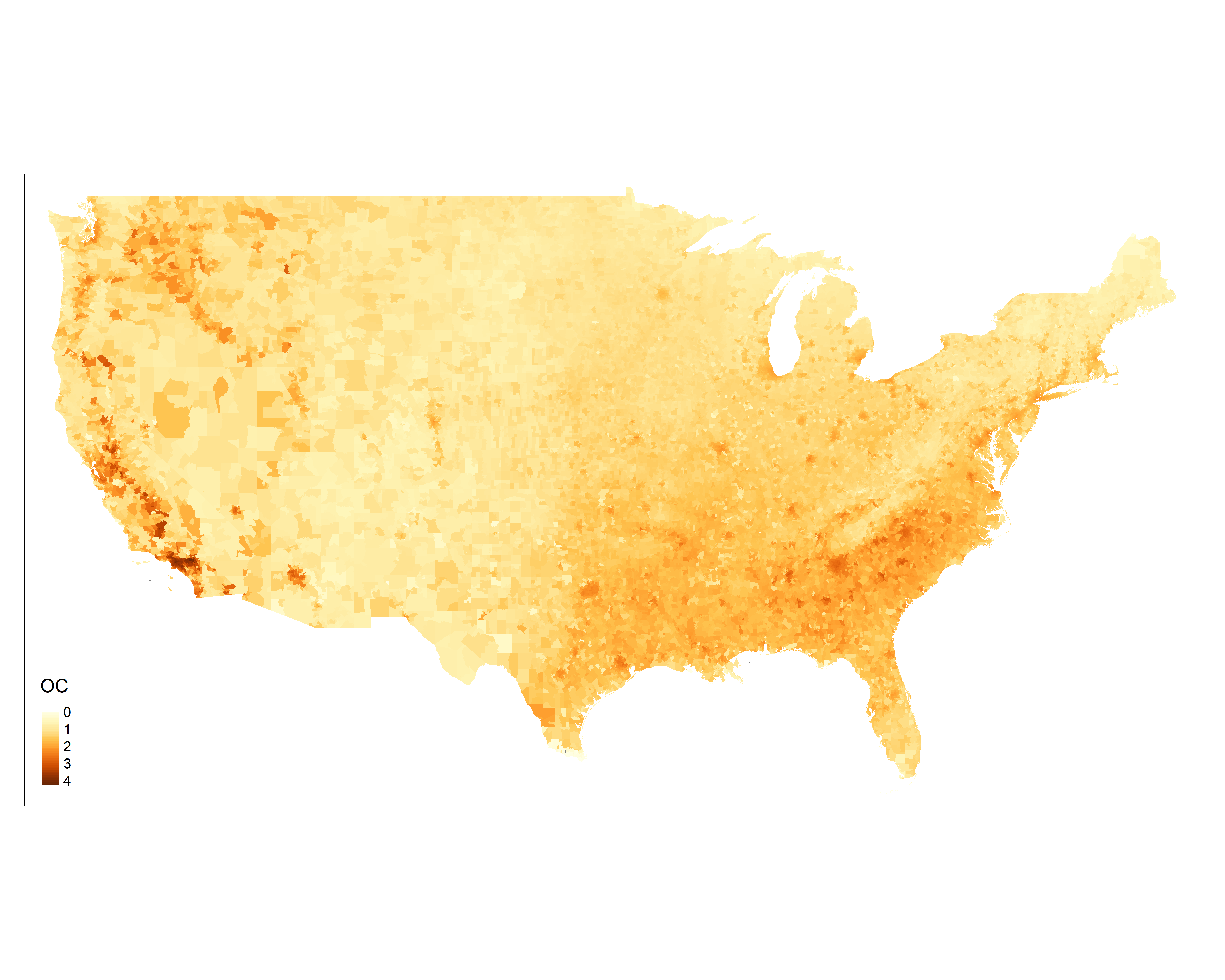

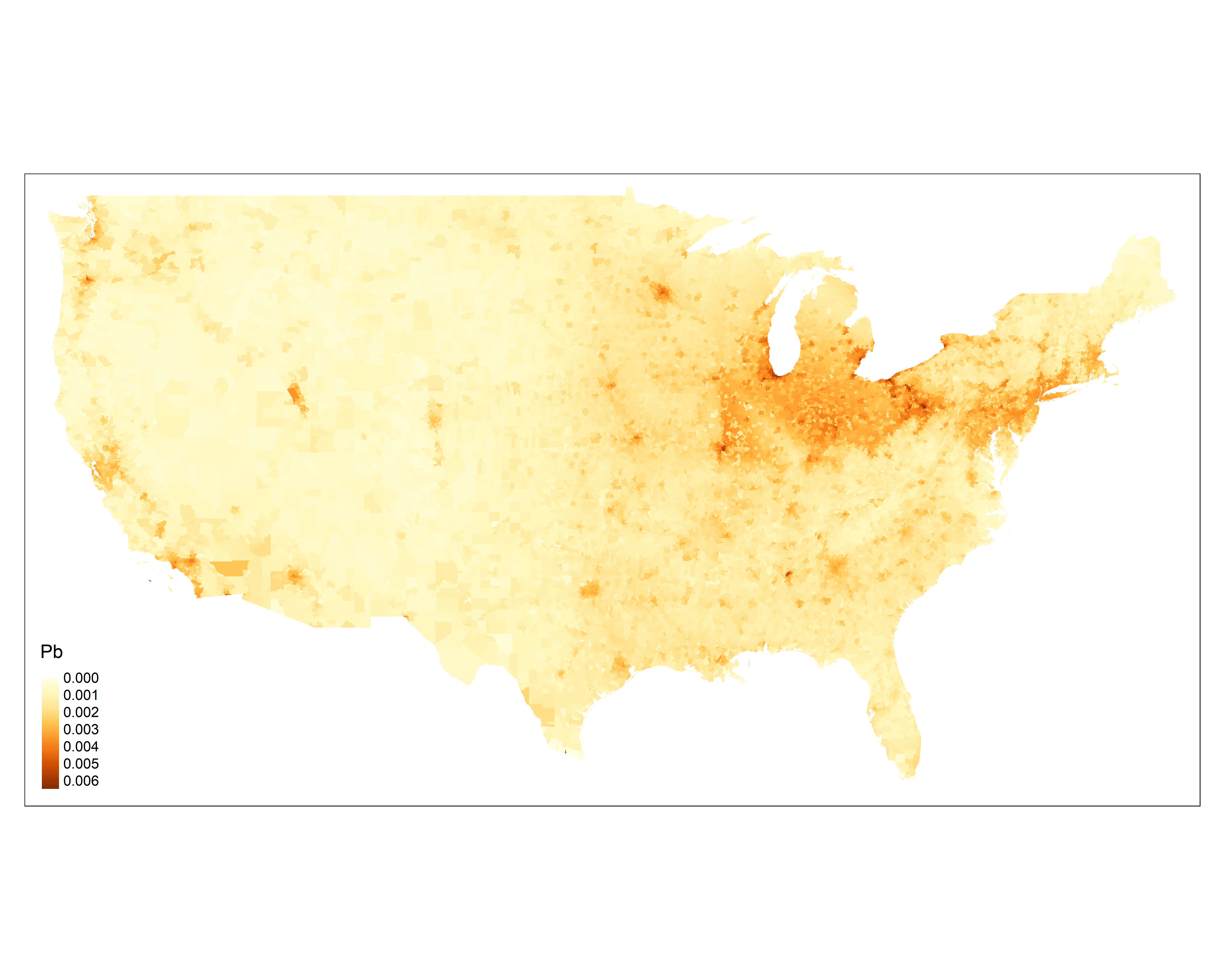

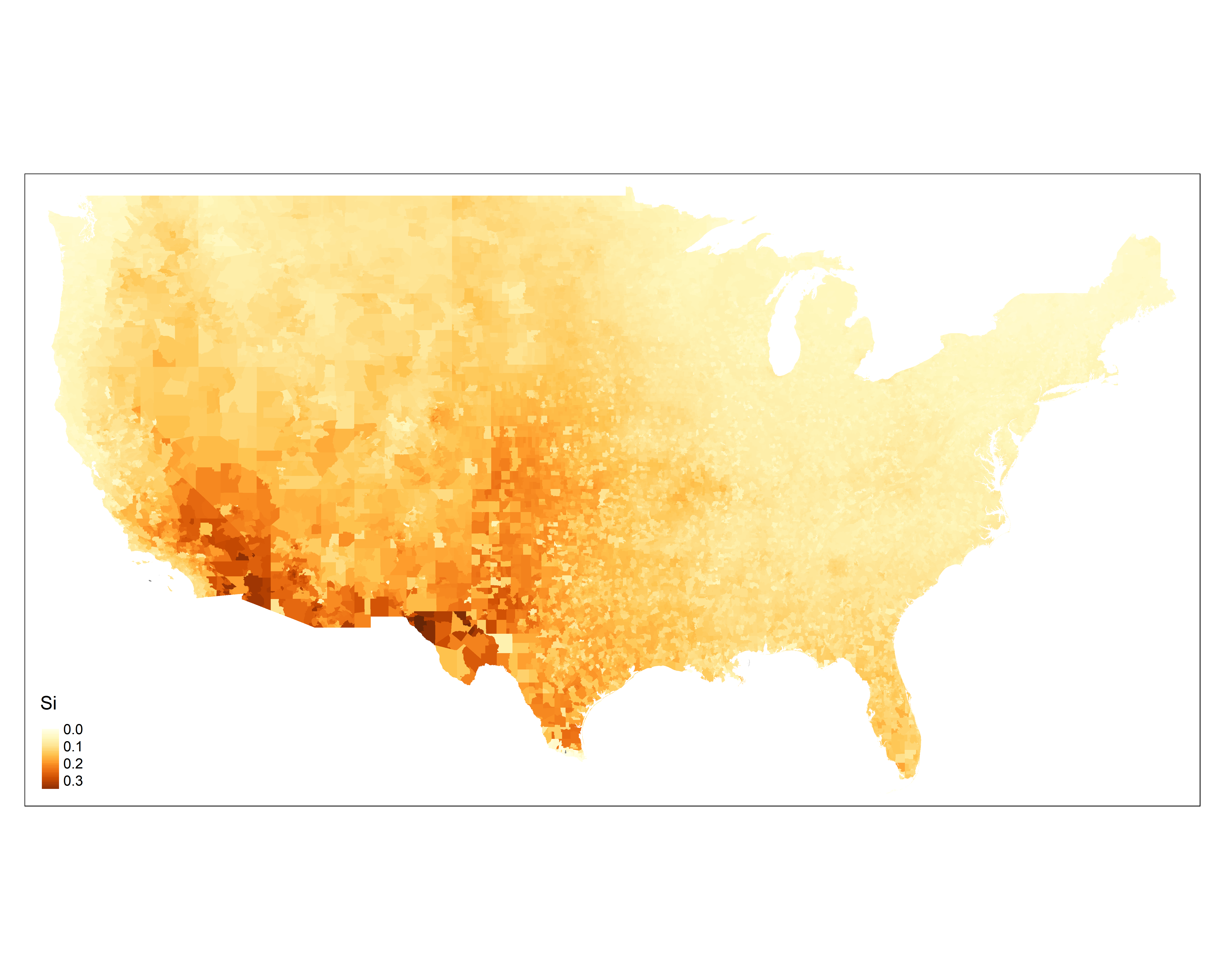

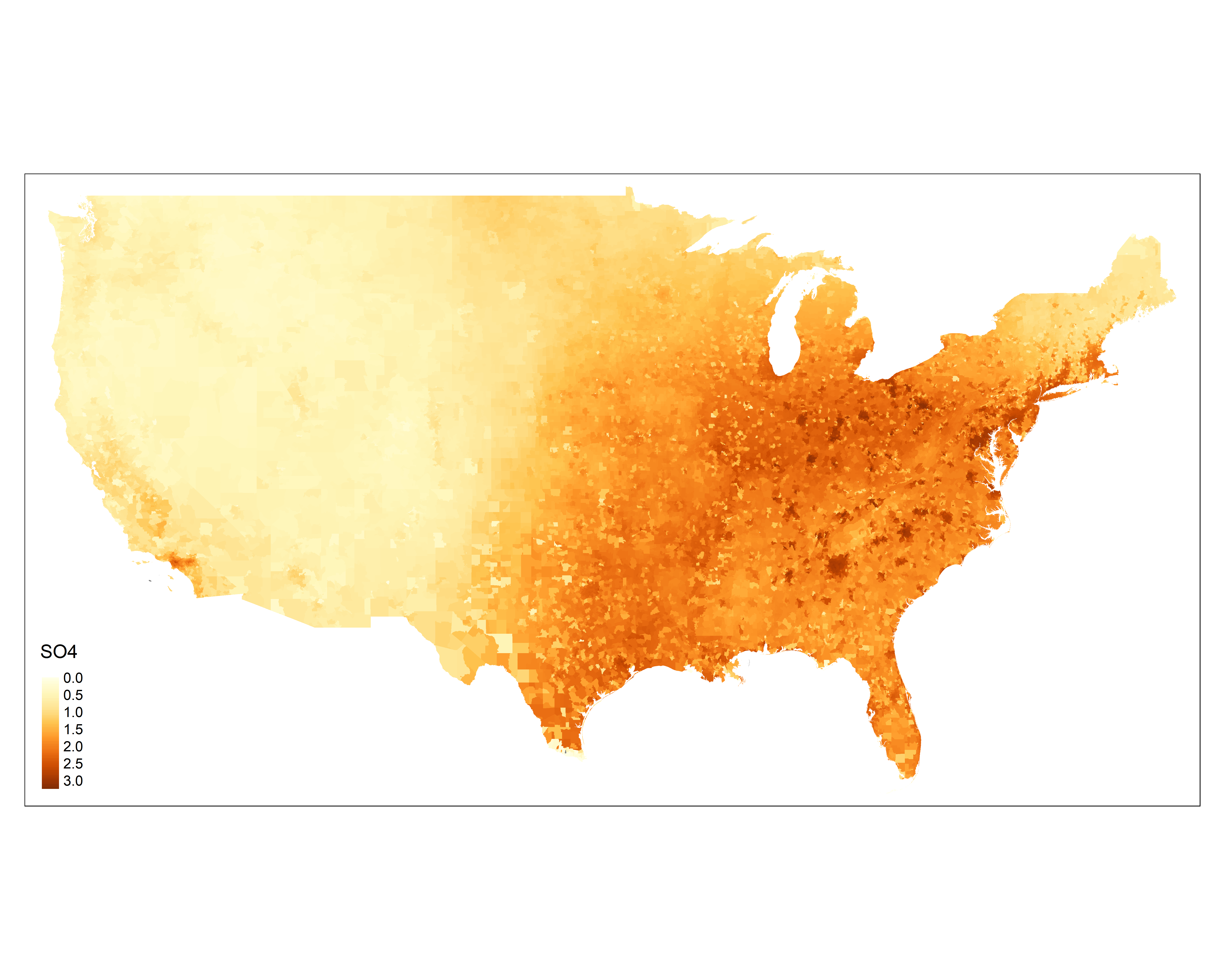

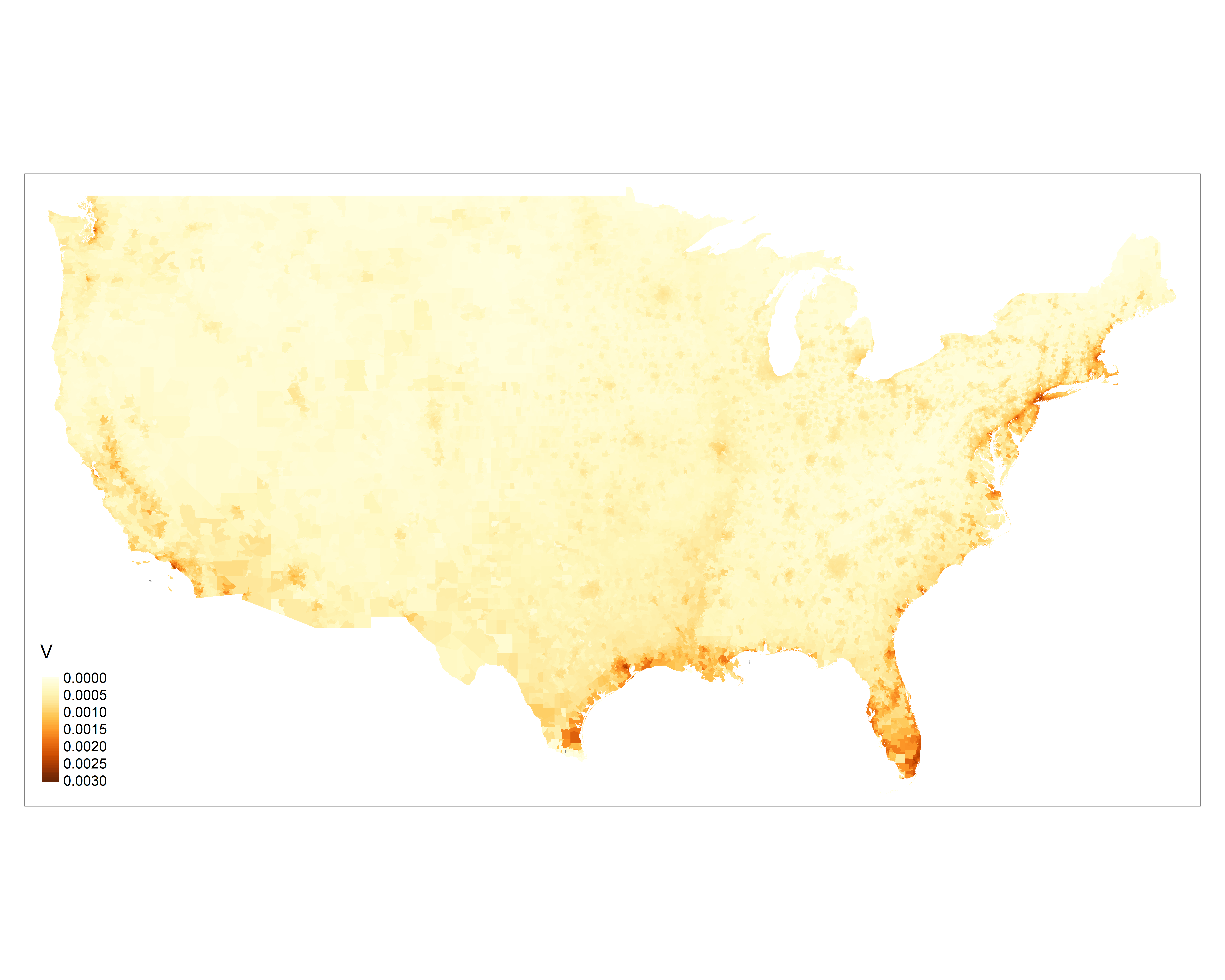

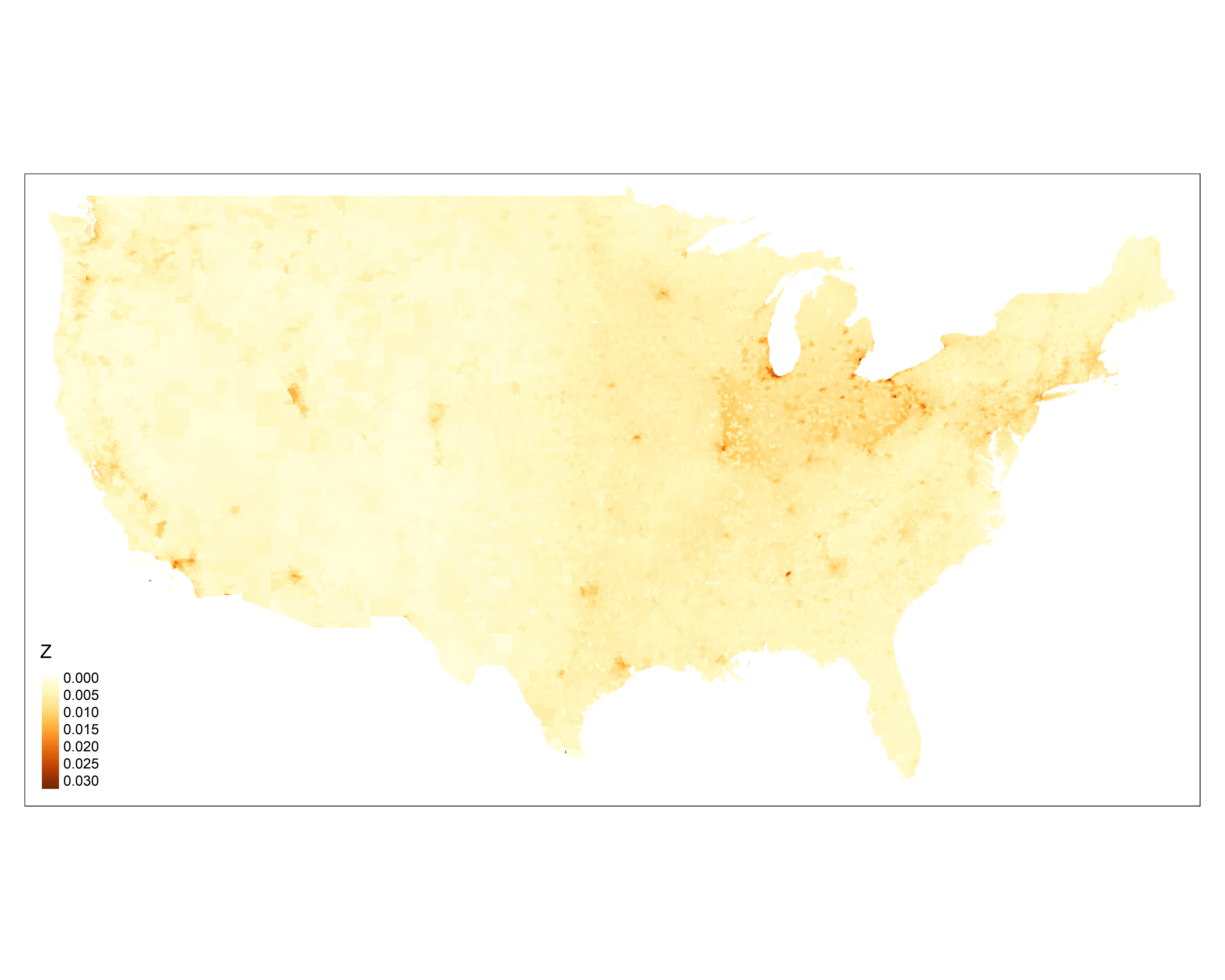


Figure S2: The correlation matrix among PM_2.5_ mass and 15 PM_2.5_ components. Pearson correlation coefficients were reported here.


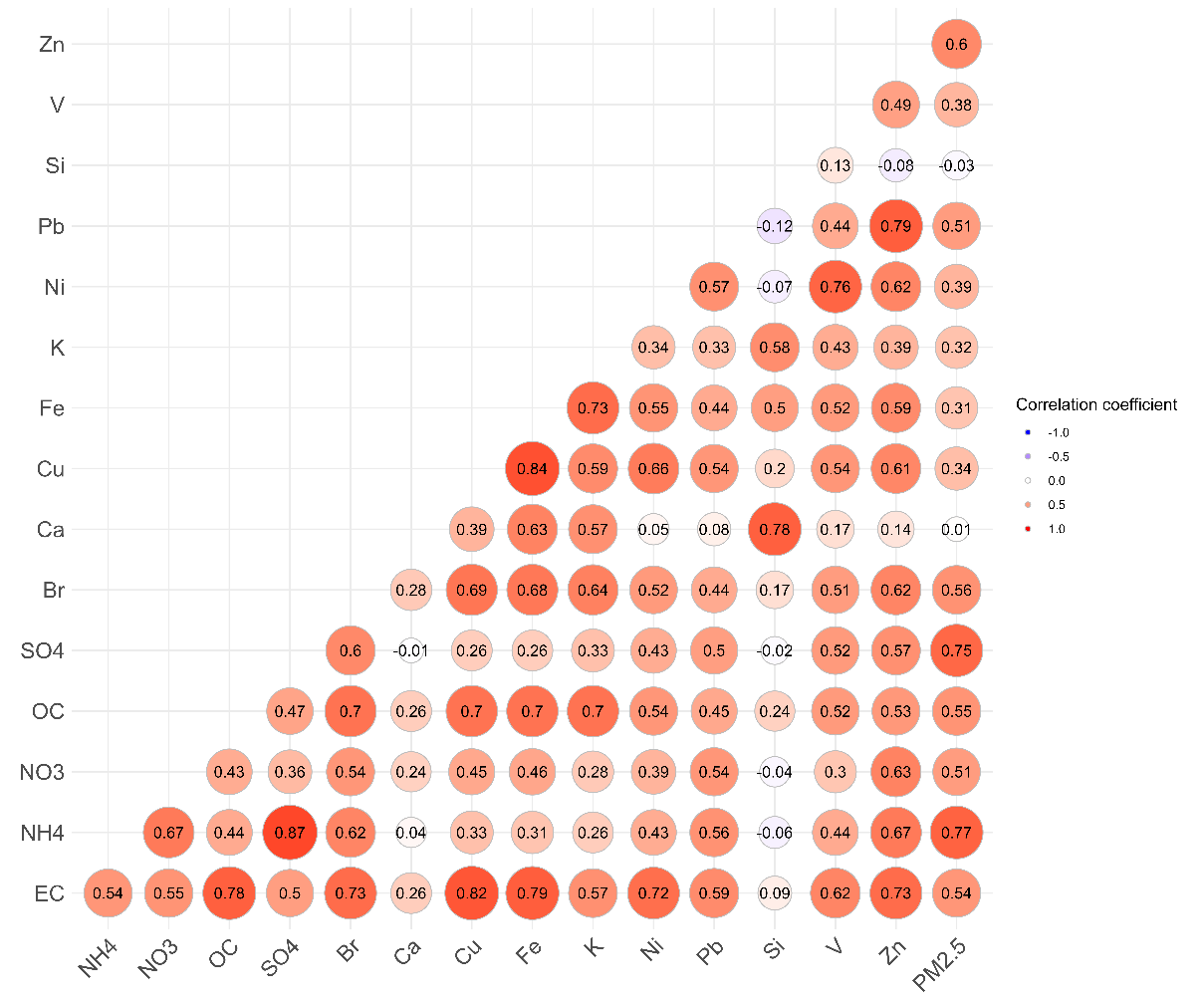


Figure S3: The annual mean concentrations of PM_2.5_ mass and PM_2.5_ components. The units for concentrations are μg/m^3^ for major mass contributors (EC, OC, NH_4_^+^, NO_3_^-^, SO4^2-^) and PM_2.5_ mass; and pg/m^3^ for trace elements (Br, Ca, Cu, Fe, K, Ni, Pb, Si, V, Zn)


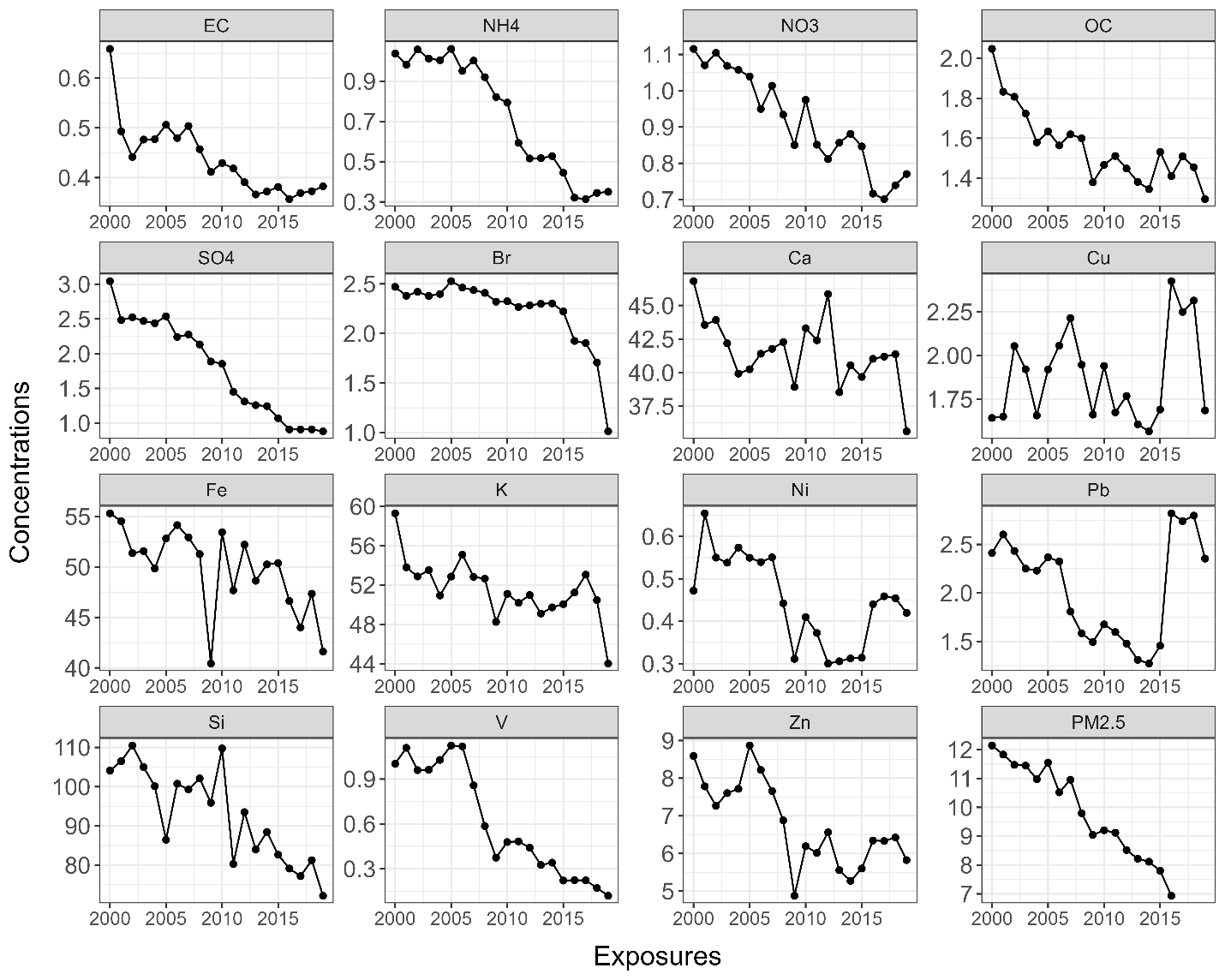


Figure S4: Hazard ratios (HRs) along with the 95% confidence intervals of dementia or Alzheimer’s disease associated with per IQR increase in annual mean concentrations of PM_2.5_ mass and PM_2.5_ components. HRs were estimated through single pollutant models.


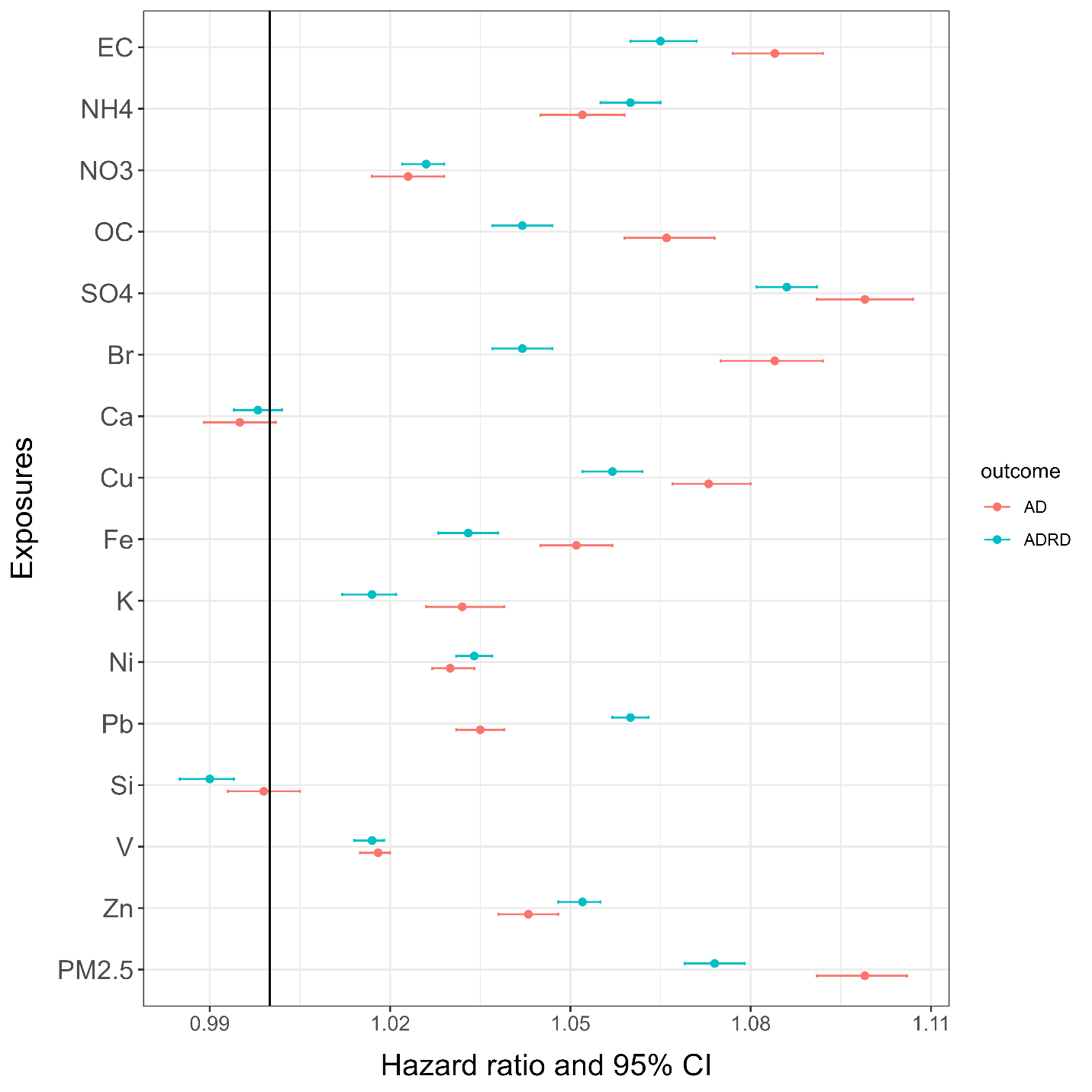


Figure S5: Hazard ratios (HRs) along with the 95% confidence intervals of dementia or Alzheimer’s disease associated with per IQR increase in annual mean concentrations of PM_2.5_ components. HRs were estimated through multi-pollutant models.


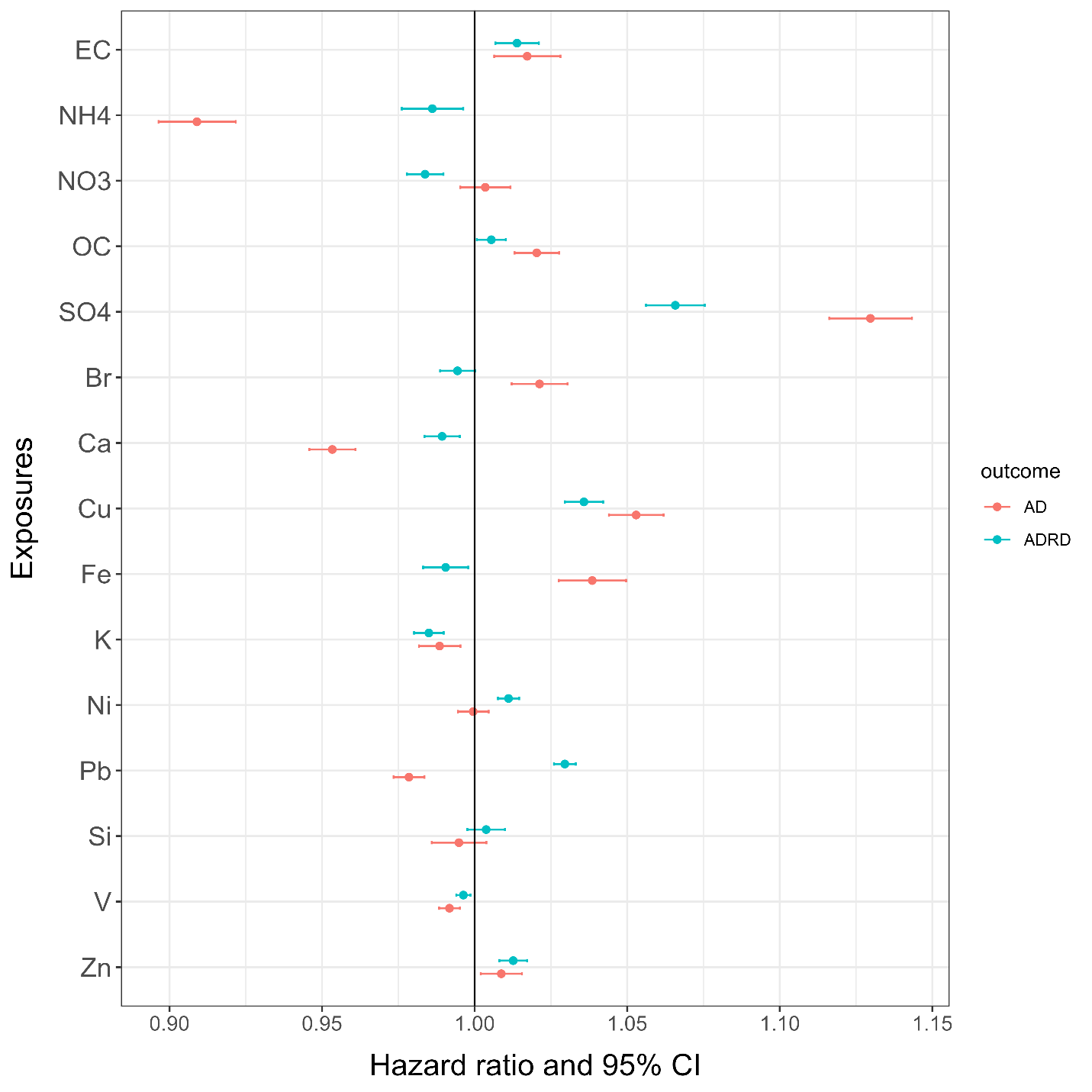


Figure S6: The weights (shown as percentages) and the calculated rate ratios (shown as bars) for each PM_2.5_ component from both WQS and qgcomp models among non-movers. Red dashed line marks the calculated rate ratio when weight = 1/15, which is the threshold for identifying potentially strong contributors among all PM_2.5_ components.

Qgcomp:


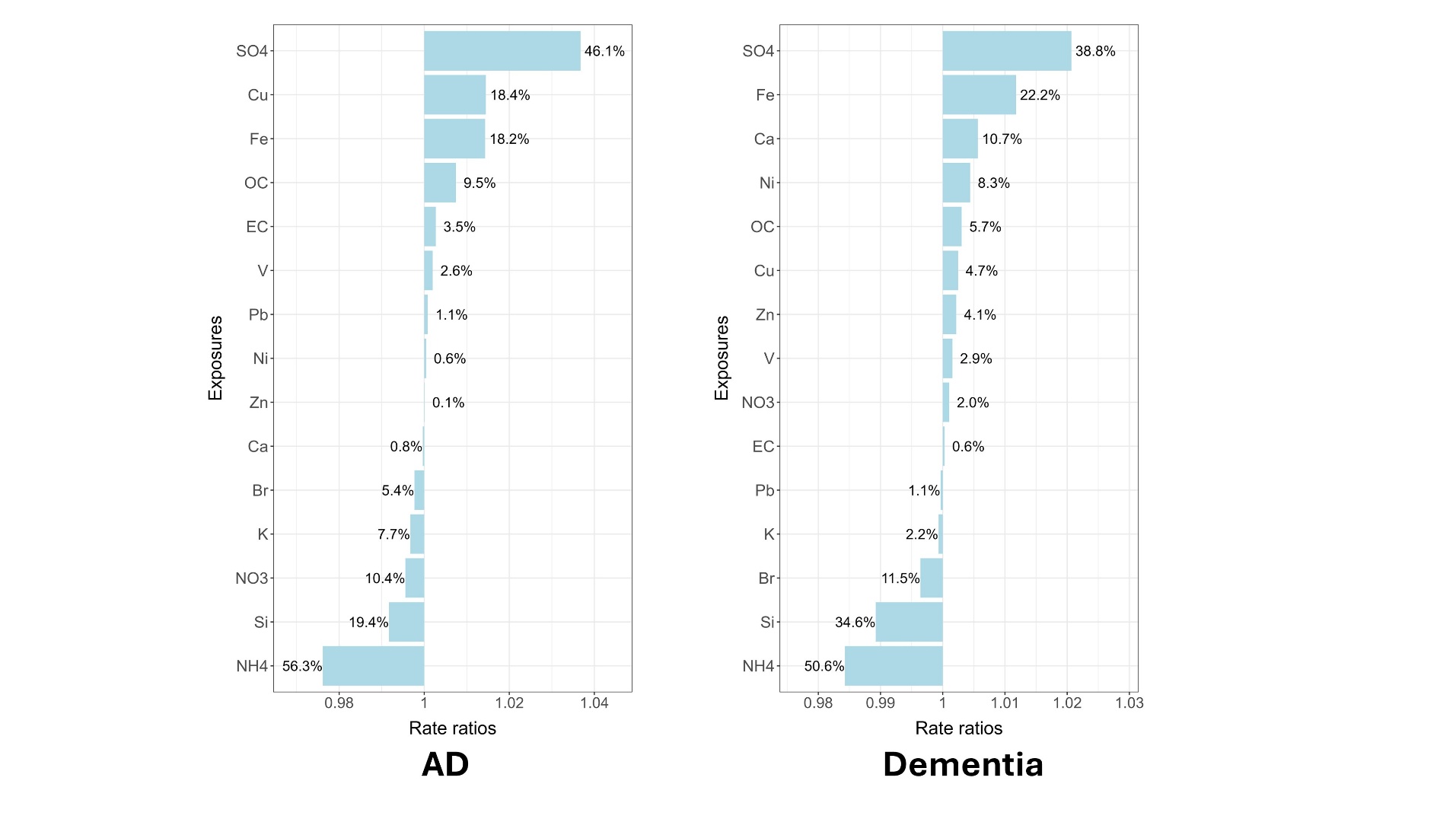


WQS:


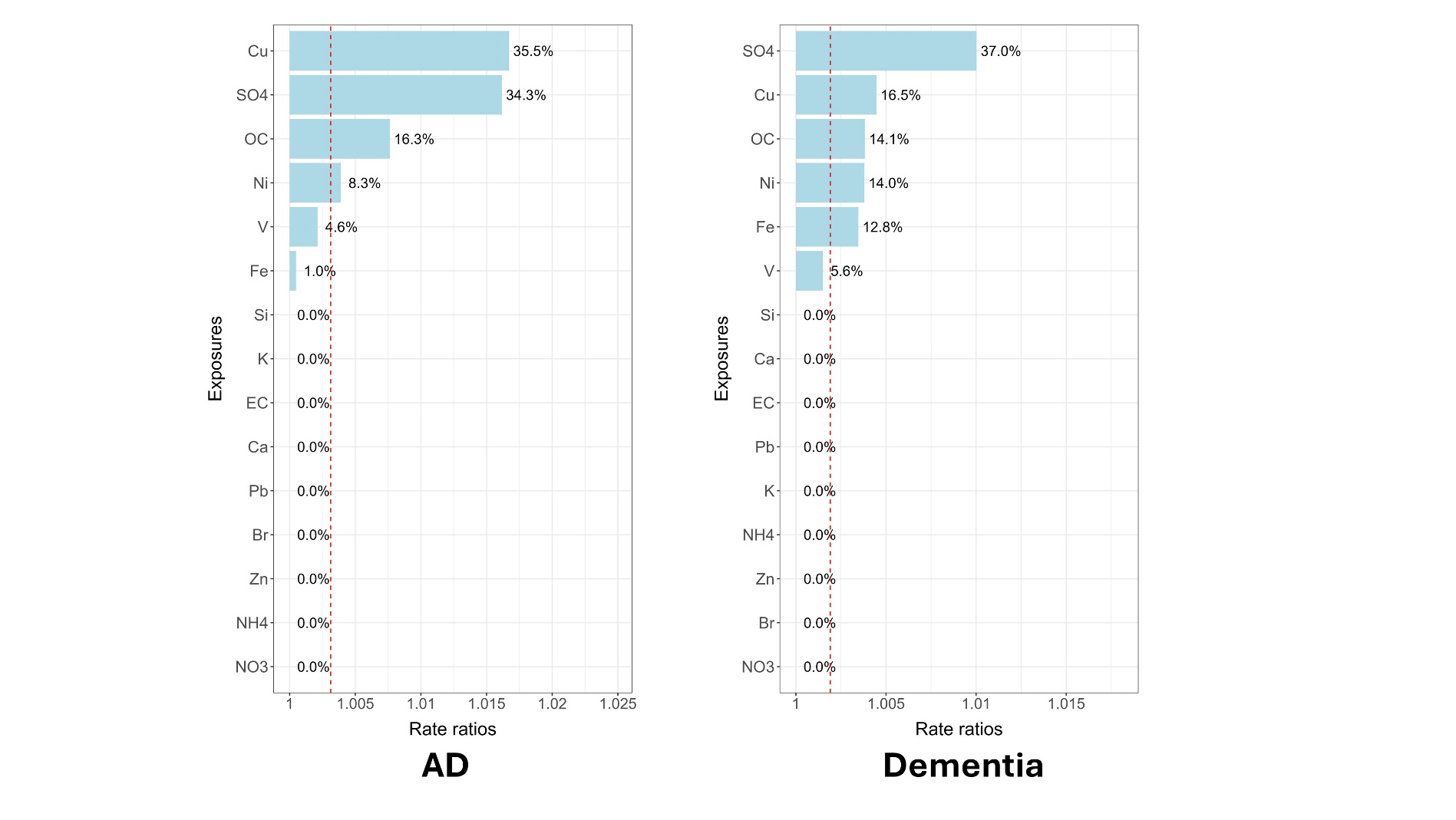


Figure S7: The weights (shown as percentages) and the calculated rate ratios (shown as bars) for each PM_2.5_ component from both WQS and qgcomp models with 3-year clean period and 4-year (same year and up to 3 years before) moving average exposures. Red dashed line marks the calculated rate ratio when weight = 1/15, which is the threshold for identifying potentially strong contributors among all PM_2.5_ components.

Qgcomp:


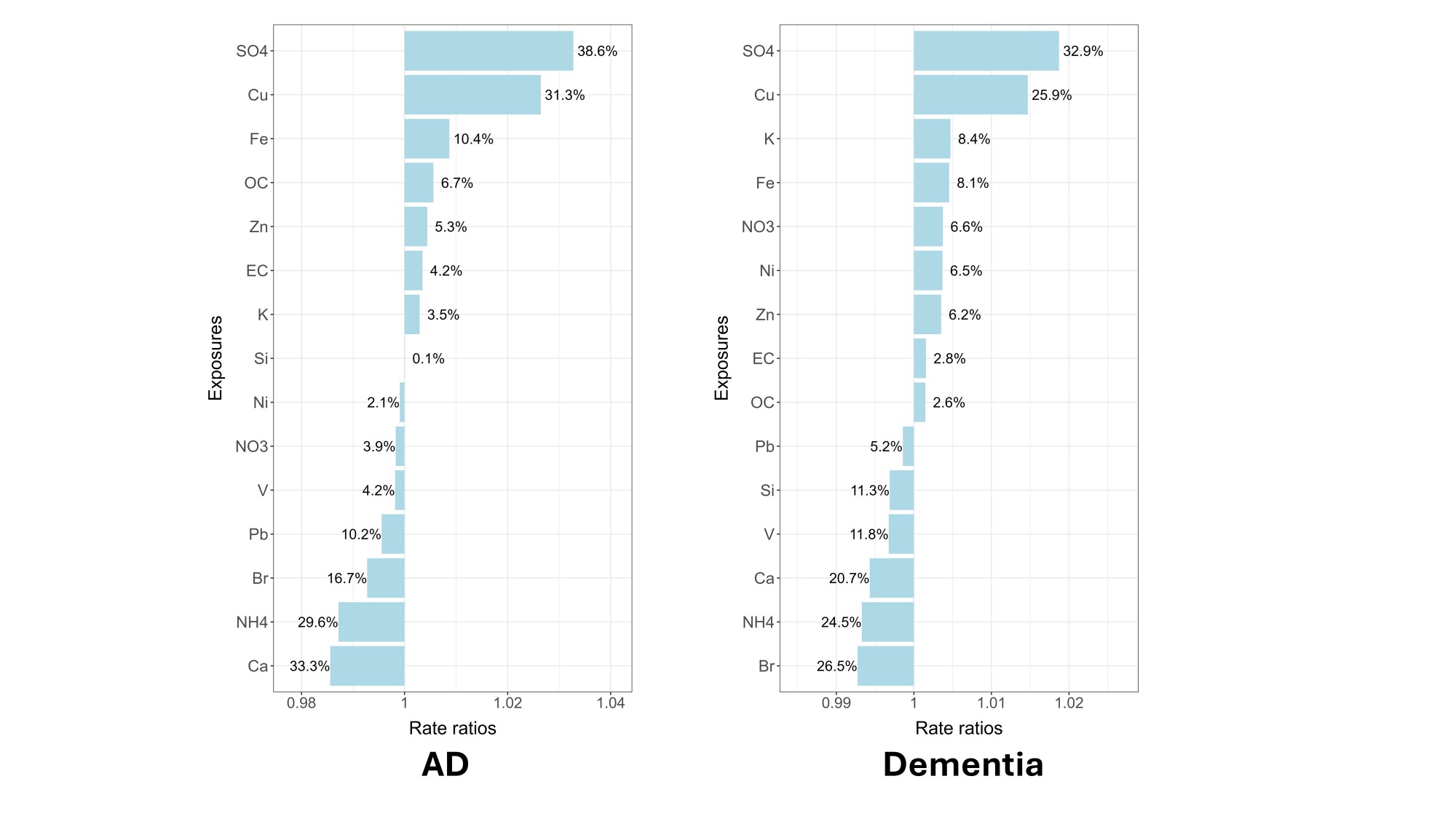


WQS:

**
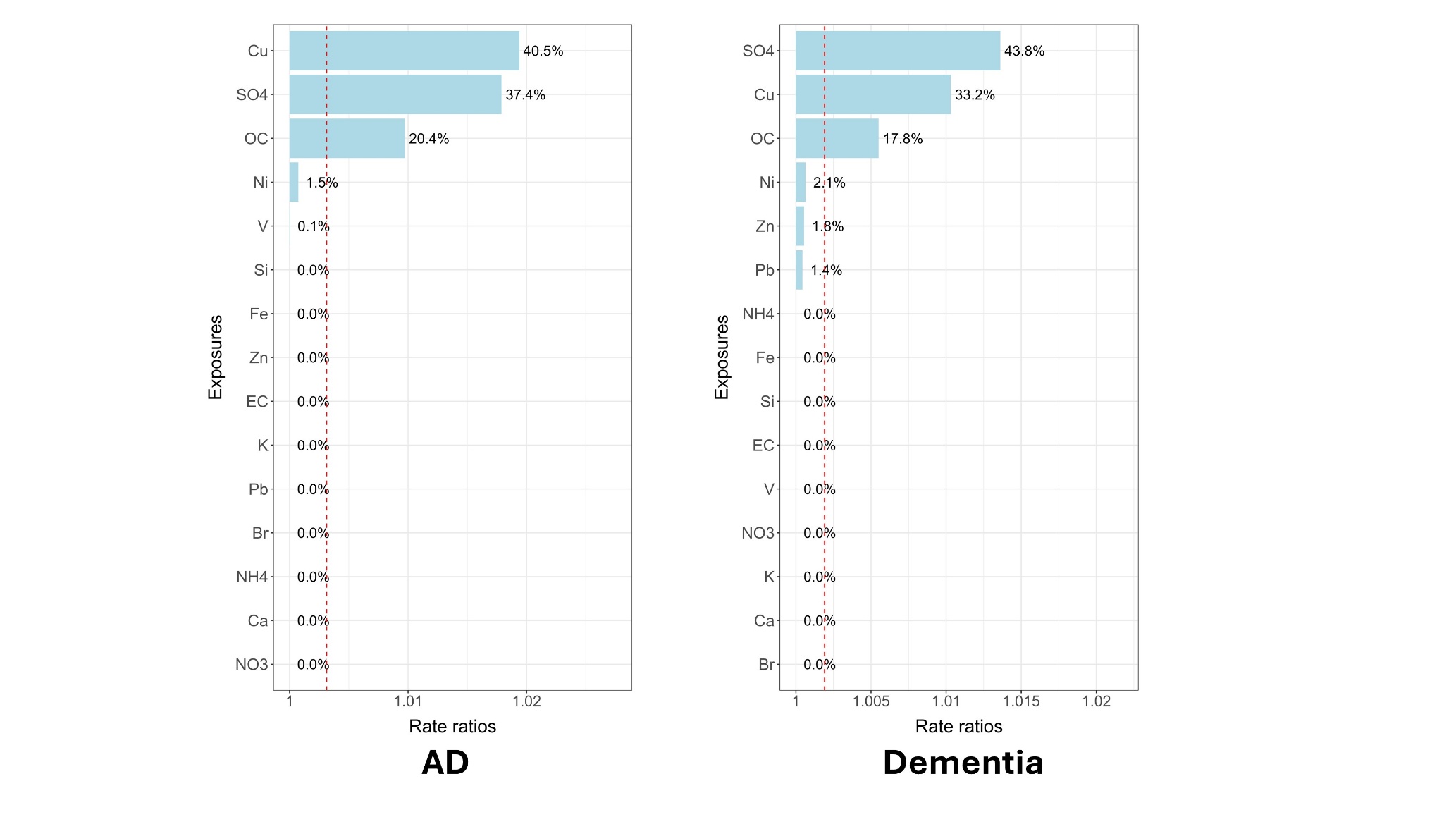
**

Table S1: ICD code associated with dementia / AD claim.

|  | AD | Dementia |
| --- | --- | --- |
| ICD-9 | DX 331.0 (any DX on the claim) | DX 331.0, 331.11, 331.19, 331.2, 331.7, 290.0, 290.10, 290.11, 290.12, 290.13, 290.20, 290.21, 290.3, 290.40, 290.41, 290.42, 290.43, 294.0, 294.10, 294.11, 294.20, 294.21, 294.8, 797 (any DX on the claim) |
| ICD-10 | DX G30.0, G30.1, G30.8, G30.9 (any DX on the claim) | DX F01.50, F01.51, F02.80, F02.81, F03.90, F03.91, F04, G13.2, G13.8, F05, F06.1, F06.8, G30.0, G30.1, G30.8, G30.9, G31.1, G31.2, G31.01, G31.09, G91.4, G94, R41.81, R54 (any DX on the claim) |

Table S2: The weights and the calculated rate ratios of each PM_2.5_ component for Alzheimer’s diseases / dementia from WQS and qgcomp models.

|  | Calculated rate ratios | | | | Weights | | | |
| --- | --- | --- | --- | --- | --- | --- | --- | --- |
|  | qgcomp | | WQS | | qgcomp | | WQS | |
| Exposure | AD | Dementia | AD | Dementia | AD | Dementia | AD | Dementia |
| EC | 1.004 | 1.001 | 1 | 1 | 0.050 | 0.024 | 0 | 0 |
| NH_4_^+^ | 0.987 | 0.994 | 1 | 1 | -0.310 | -0.216 | 0 | 0 |
| NO_3_^-^ | 0.995 | 1.001 | 1 | 1 | -0.121 | 0.015 | 0 | 0 |
| OC | 1.007 | 1.003 | 1.010 | 1.007 | 0.091 | 0.047 | 0.221 | 0.240 |
| SO4^2-^ | 1.034 | 1.018 | 1.017 | 1.013 | 0.406 | 0.328 | 0.367 | 0.440 |
| Br | 0.992 | 0.992 | 1 | 1 | -0.193 | -0.281 | 0 | 0 |
| Ca | 0.991 | 0.998 | 1 | 1 | -0.226 | -0.078 | 0 | 0 |
| Cu | 1.026 | 1.014 | 1.019 | 1.008 | 0.312 | 0.265 | 0.405 | 0.264 |
| Fe | 1.007 | 1.004 | 1 | 1 | 0.080 | 0.074 | 0 | 0 |
| K | 1.001 | 1.005 | 1 | 1 | 0.016 | 0.084 | 0 | 0 |
| Ni | 0.999 | 1.004 | 1 | 1.002 | -0.032 | 0.067 | 0.006 | 0.055 |
| Pb | 0.999 | 0.998 | 1 | 1 | -0.020 | -0.091 | 0 | 0 |
| Si | 0.997 | 0.994 | 1 | 1 | -0.074 | -0.222 | 0 | 0 |
| V | 0.999 | 0.997 | 1 | 1 | -0.024 | -0.112 | 0 | 0 |
| Zn | 1.004 | 1.005 | 1 | 1 | 0.045 | 0.095 | 0 | 0 |

Table S3: Hazard ratios (HRs) along with the 95% confidence intervals of dementia or Alzheimer’s disease associated with per IQR increase in annual mean concentrations of PM_2.5_ components, from both single pollutant and multi-pollutant Cox models.

|  | Single pollutant | | Multi-pollutant | |
| --- | --- | --- | --- | --- |
|  | Demetia | AD | Dementia | AD |
| EC | 1.065 (1.060, 1.071) | 1.084 (1.077, 1.092) | 1.014 (1.007, 1.021) | 1.017 (1.006, 1.028) |
| NH_4_^+^ | 1.060 (1.055, 1.065) | 1.052 (1.045, 1.059) | 0.986 (0.976, 0.996) | 0.909 (0.896, 0.922) |
| NO_3_^-^ | 1.026 (1.022, 1.029) | 1.023 (1.017, 1.029) | 0.984 (0.978, 0.990) | 1.003 (0.995, 1.012) |
| OC | 1.042 (1.037, 1.047) | 1.066 (1.059, 1.074) | 1.005 (1.001, 1.010) | 1.020 (1.013, 1.028) |
| SO4^2-^ | 1.086 (1.081, 1.091) | 1.099 (1.091, 1.107) | 1.066 (1.056, 1.075) | 1.130 (1.116, 1.143) |
| Br | 1.042 (1.037, 1.047) | 1.084 (1.075, 1.092) | 0.994 (0.989, 1.000) | 1.021 (1.012, 1.030) |
| Ca | 0.998 (0.994, 1.002) | 0.995 (0.989, 1.001) | 0.989 (0.984, 0.995) | 0.953 (0.946, 0.961) |
| Cu | 1.057 (1.052, 1.062) | 1.073 (1.067, 1.080) | 1.036 (1.030, 1.042) | 1.053 (1.044, 1.062) |
| Fe | 1.033 (1.028, 1.038) | 1.051 (1.045, 1.057) | 0.990 (0.983, 0.998) | 1.039 (1.028, 1.050) |
| K | 1.017 (1.012, 1.021) | 1.032 (1.026, 1.039) | 0.985 (0.980, 0.990) | 0.989 (0.982, 0.995) |
| Ni | 1.034 (1.031, 1.037) | 1.030 (1.027, 1.034) | 1.011 (1.008, 1.015) | 1.000 (0.995, 1.005) |
| Pb | 1.060 (1.057, 1.063) | 1.035 (1.031, 1.039) | 1.030 (1.026, 1.033) | 0.978 (0.973, 0.984) |
| Si | 0.990 (0.985, 0.994) | 0.999 (0.993, 1.005) | 1.004 (0.998, 1.010) | 0.995 (0.986, 1.004) |
| V | 1.017 (1.014, 1.019) | 1.018 (1.015, 1.020) | 0.996 (0.994, 0.999) | 0.992 (0.988, 0.995) |
| Zn | 1.052 (1.048, 1.055) | 1.043 (1.038, 1.048) | 1.013 (1.008, 1.017) | 1.009 (1.002, 1.016) |
| PM_2.5_ | 1.074 (1.069, 1.079) | 1.099 (1.091, 1.106) |  |  |

Table S4: Cumulative associations between PM_2.5_ components and Alzheimer’s diseases / dementia in rate ratios estimated from both WQS and qgcomp models under different settings.

| Models | Mover status | Clean & lag period | AD | Dementia |
| --- | --- | --- | --- | --- |
| qgcomp |  |  |  |  |
|  | All | 5yr | 1.041 (1.039, 1.042) | 1.027 (1.025, 1.028) |
|  | Non-movers | 5yr | 1.036 (1.034, 1.038) | 1.022 (1.020, 1.023) |
|  | All | 3yr | 1.041 (1.039, 1.042) | 1.027 (1.025, 1.028) |
| WQS |  |  |  |  |
|  | All | 5yr | 1.048 (1.047, 1.049) | 1.029 (1.028, 1.030) |
|  | Non-movers | 5yr | 1.048 (1.046, 1.049) | 1.027 (1.026, 1.028) |
|  | All | 3yr | 1.049 (1.047, 1.050) | 1.031 (1.031, 1.032) |

Table S5: The weights and the calculated rate ratios of each PM_2.5_ component for Alzheimer’s diseases / dementia from WQS and qgcomp models among non-movers.

|  | Calculated rate ratios | | | | Weights | | | |
| --- | --- | --- | --- | --- | --- | --- | --- | --- |
|  | qgcomp | | WQS | | qgcomp | | WQS | |
| Exposure | AD | Dementia | AD | Dementia | AD | Dementia | AD | Dementia |
| EC | 1.003 | 1 | 1 | 1 | 0.035 | 0.006 | 0 | 0 |
| NH_4_^+^ | 0.976 | 0.984 | 1 | 1 | -0.563 | -0.506 | 0 | 0 |
| NO_3_^-^ | 0.996 | 1.001 | 1 | 1 | -0.104 | 0.020 | 0 | 0 |
| OC | 1.007 | 1.003 | 1.008 | 1.004 | 0.095 | 0.057 | 0.163 | 0.141 |
| SO4^2-^ | 1.037 | 1.021 | 1.016 | 1.010 | 0.461 | 0.388 | 0.343 | 0.370 |
| Br | 0.998 | 0.996 | 1 | 1 | -0.054 | -0.115 | 0 | 0 |
| Ca | 1 | 1.006 | 1 | 1 | -0.008 | 0.107 | 0 | 0 |
| Cu | 1.014 | 1.002 | 1.017 | 1.004 | 0.184 | 0.047 | 0.355 | 0.165 |
| Fe | 1.014 | 1.012 | 1 | 1.003 | 0.182 | 0.222 | 0.010 | 0.128 |
| K | 0.997 | 0.999 | 1 | 1 | -0.077 | -0.022 | 0 | 0 |
| Ni | 1 | 1.004 | 1.004 | 1.004 | 0.006 | 0.083 | 0.083 | 0.140 |
| Pb | 1.001 | 1 | 1 | 1 | 0.011 | -0.011 | 0 | 0 |
| Si | 0.992 | 0.989 | 1 | 1 | -0.194 | -0.346 | 0 | 0 |
| V | 1.002 | 1.002 | 1.002 | 1.002 | 0.026 | 0.029 | 0.046 | 0.056 |
| Zn | 1 | 1.002 | 1 | 1 | 0.001 | 0.041 | 0 | 0 |

Table S6: The weights and the calculated rate ratios of each PM_2.5_ component for Alzheimer’s diseases / dementia from WQS and qgcomp models with 3-year clean period and 4-year (same year and up to 3 years before) moving average exposures.

|  | | Calculated rate ratios | | | | Weights | | | |
| --- | --- | --- | --- | --- | --- | --- | --- | --- | --- |
|  | | qgcomp | | WQS | | qgcomp | | WQS | |
| Exposure | | AD | Dementia | AD | Dementia | AD | Dementia | AD | Dementia |
| EC | | 1.003 | 1.002 | 1 | 1 | 0.042 | 0.028 | 0 | 0 |
| NH_4_^+^ | | 0.987 | 0.993 | 1 | 1 | -0.296 | -0.245 | 0 | 0 |
| NO_3_^-^ | | 0.998 | 1.004 | 1 | 1 | -0.039 | 0.066 | 0 | 0 |
| OC | | 1.006 | 1.001 | 1.01 | 1.006 | 0.067 | 0.026 | 0.204 | 0.178 |
| SO4^2-^ | | 1.033 | 1.019 | 1.018 | 1.014 | 0.386 | 0.329 | 0.374 | 0.438 |
| Br | | 0.993 | 0.993 | 1 | 1 | -0.167 | -0.265 | 0 | 0 |
| Ca | | 0.986 | 0.994 | 1 | 1 | -0.333 | -0.207 | 0 | 0 |
| Cu | 1.026 | | 1.015 | 1.019 | 1.01 | 0.313 | 0.259 | 0.405 | 0.332 |
| Fe | 1.009 | | 1.005 | 1 | 1 | 0.104 | 0.081 | 0 | 0 |
| K | 1.003 | | 1.005 | 1 | 1 | 0.035 | 0.084 | 0 | 0 |
| Ni | 0.999 | | 1.004 | 1.001 | 1.001 | -0.021 | 0.065 | 0.015 | 0.021 |
| Pb | 0.996 | | 0.999 | 1 | 1 | -0.102 | -0.052 | 0 | 0.014 |
| Si | 1 | | 0.997 | 1 | 1 | 0.001 | -0.113 | 0 | 0 |
| V | 0.998 | | 0.997 | 1 | 1 | -0.042 | -0.118 | 0.001 | 0 |
| Zn | 1.004 | | 1.004 | 1 | 1.001 | 0.053 | 0.062 | 0 | 0.018 |

**Data availability statement**

The PM_2.5_ data that support the findings of this study are publicly available from https://doi.org/10.7927/0rvr-4538.

PM_2.5_ components EC, NH_4_^+^, NO_3_^-^, OC, SO_4_^2-^(50 m^2^ urban and 1 km^2^ nonurban, annual level, 2000-2019) data that support the findings of this study are publicly available from: https://sedac.ciesin.columbia.edu/data/set/aqdh-pm2-5-component-ec-nh4-no3-oc-so4-50m-1km-contiguous-us-2000-2019

PM_2.5_ trace elements (50 m^2^ urban and 1 km^2^ nonurban, annual level, 2000-2019) data that support the findings of this study are publicly available from: https://sedac.ciesin.columbia.edu/data/set/aqdh-pm2-5-component-trace-elements-50m-1km-contiguous-us-2000-2019

Behavioral risk factors are publicly available from https://www.cdc.gov/brfss/annual_data/annual_data.html.

SES data are publicly available from https://www.census.gov/data/datasets/2000/dec/summary-file-3.html, https://www.census.gov/data/datasets/2010/dec/summary-file-1.html, and https://www.census.gov/data/developers/data-sets/acs-1year.html.

Health-care capacity data are available from https://data.hrsa.gov/topics/health-workforce/ahrf.

The rules governing the main Medicare dataset used in this study prohibit any sharing of the health datasets being used here, restricted by our Data Use Agreement with the US Centers for Medicare & Medicaid Services. Academic and non-profit researchers who are interested in using Medicare data should contact the US Centers for Medicare & Medicaid Services directly to obtain their own datasets upon completion of a Data Use Agreement.
